# Investigation of Aggregation Induced Emission Mechanism of Tetrabenzoheptafulvalene Derivative by Spin‐Flip Time‐Dependent Density Functional Theory (SF‐TDDFT)

**DOI:** 10.1002/asia.202401617

**Published:** 2025-02-26

**Authors:** Aarzoo, Ram Kinkar Roy

**Affiliations:** ^1^ Dept. of Chemistry BITS-PILANI Pilani Campus Rajasthan India

**Keywords:** Aggregation-induced emission, spin-flip time-dependent density functional theory, minimum energy conical intersection, oscillator strength, minimum energy gap

## Abstract

This study explores the mechanism of aggregation‐induced emission (AIE) in the tetrabenzoheptafulvalene derivative, 10,10′,11,11′‐tetrahydro‐5,5′‐bidibenzo[a,d][7]annulenylidene (abbreviated as THBDBA) in tetrahydrofuran (THF) solution. THBDBA is AIE‐active because in THF solution, it emits significantly less emission (or almost non‐emissive) and the fluorescence quantum yield increases by 230 times in aggregate state. We adopted spin‐flip time‐dependent density functional theory (SF‐TDDFT), widely acknowledged for its ability to locate the conical intersection (CI) in medium to large‐sized molecules (due to its balanced and reliable description of both ground and excited states and ability to capture double excitation and multireference characters at low computational cost). The functional used is long‐range corrected ωPBEh (i. e., LRC‐ωPBEh). The strategies used are the excited state deactivation processes by taking into account the S_1_/S_0_ surface crossing, referred to as the ‘minimum energy conical intersection’ (MECI). Reduction of oscillator strength near the minimum energy gap (MEG) structure or CI is also another parameter used to study fluorescence quenching. For the monomer (i. e., in solution), our findings reveal a significant reduction in oscillator strength (*f*) for de‐excitation near the MEG structure and CI, which led us to conclude that in solution, the flapping motion of the phenyl rings plays a vital role to reach the CI. In a smaller scale, a dimer system was chosen to represent the aggregate state. The higher energy gap as well as higher *f‐*value at MEG structure with just the model dimer system indicates that in the actual aggregate (or the crystal) the MECI might be absent. This is because in the aggregate the flapping motion of the phenyl rings will be highly restricted (because of the steric and electrostatic confinements by a large number of monomers from all sides), thereby favoring radiative transitions for energy dissipation. This study consequently elucidates the AIE mechanism of the chosen tetrabenzoheptafulvalene derivative, shedding light on its photophysical properties.

## Graphical abstract

The SF‐TDDFT study of 10,10′,11,11′‐tetrahydro‐5,5′‐bidibenzo[a,d][7]annulenylidene (a tetrabenzoheptafulvalene derivative, abbreviated as THBDBA) in THF solution reveals the cause of its AIE behavior, While in solution intramolecular vibration leads to conical intersection between S_0_ and S_1_ states (i. e., fluorescence quenching), in aggregate state restricted intramolecular vibration (RIV) causes the inhibition of fluorescence quenching (i. e., AIE).

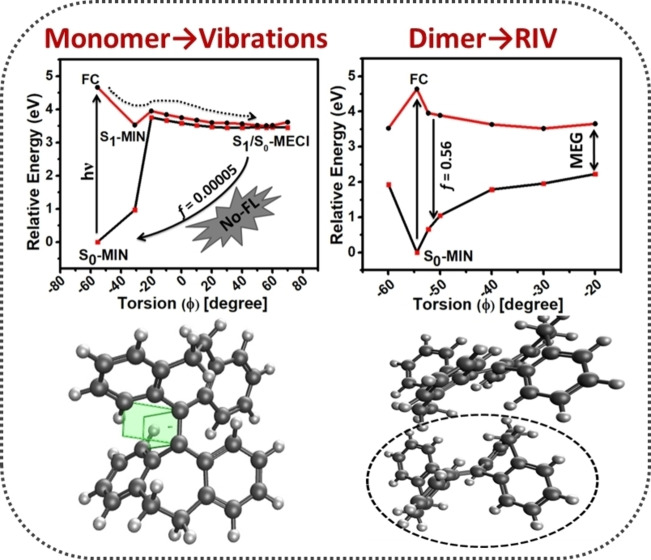

## 1 Introduction

Most organic luminogens employ planar and highly conjugated aromatic systems to obtain high quantum yields in solution, i. e., while completely solvated these compounds emit intense luminescence. However, due to their extremely planar and conjugated structures, at high concentrations or in aggregate state, the fierce fluorescence of most organic luminogens is reduced or completely turned off. This phenomenon is termed ‘Aggregation‐caused quenching’ (ACQ).^[1]^ In 2001, Tang et al. observed an opposite phenomenon of ACQ in which the molecules are non‐emissive (or less emissive) at the molecular state but highly emissive at the aggregated state. They introduced the concept of ‘Aggregation‐induced Emission’ (AIE) to explain this phenomenon, and the first molecule that witnessed this phenomenon was methyl‐1,2,3,4,5‐pentaphenylsilole.^[2]^ Molecular motions are abundant in nature, playing fundamental roles at all levels, from the microscopic to the macroscopic scale.[^3^, ^4^] AIE luminogens (AIEgens) have recently shown exceptional features in investigating molecular motions, making them an ideal visualisation model.[^5^, ^6^, ^7^, ^8^]

There have been persistent efforts to clarify thoroughly the emission mechanism of AIEgens and now it is generally accepted that the root cause of AIE is the restriction of intra‐molecular motion (RIM). Again, RIM includes two different aspects: the restriction of intra‐molecular vibration (RIV) and the restriction of intra‐molecular rotation (RIR).[^9^, ^10^, ^11^] In particular, when the AIEgens are in a molecular state, the free active intra‐molecular motion will promote the non‐radiative decay pathway and lead to a non‐emissive state. Once they form aggregates, RIM will effectively block the non‐radiative decay pathway, leading to enhanced emission. Subsequently, several novel AIEgens have been developed for various applications, including electroluminescent devices,^[12]^ detection,^[13]^ chemical probing,^[14]^ biotechnology, and pharmacology.[^15^, ^16^, ^17^] As of today, several AIEgens have been developed, including hexaphenylsilole (HPS),^[18]^ tetraphenylethene (TPE),[^19^, ^20^] triphenylamine (TPA) and their derivatives,[^21^, ^22^, ^23^] heterocyclic compounds containing Si, N, or P,[^24^, ^25^] and compounds that included the BF_2_ hexatomic ring,[^26^, ^27^] and many others. It could be inferred that most of the organic AIEgens originate via the RIR mechanism, specifically those made up of conventional cores like aryl ethylene, TPA, TPE, HPS, etc.^[28]^ But there have been very few RIV based AIEgens until the discovery of 10,10′,11,11′‐tetrahydro‐5,5′‐bidibenzo[a,d][7]annulenylidene (THBDBA) and 5,5′‐bidibenzo[a,d][7]annulenylidene (BDBA).[^9^, ^29^] Developing novel, highly effective compounds with AIE properties and their utilization in many different fields is a prominent research topic,^[30]^ and comprehending the fundamental mechanism of AIE is vital for effective design. In addition, RIV systems are desired and necessary models for investigating molecular motion, and there is an enormous demand for new RIV based AIE cores.[^9^, ^28^, ^29^] However, because of the ambiguous molecular design, RIV‐based molecular systems are significantly less explored and still require greater variety. The RIV mechanism explains the AIE systems with non‐planar vibrators without many rotors. It is expected that, by following the RIM principle, the field of AIE systems will be significantly broadened, greatly expanding the scope of AIE research.

The RIM and related models are theoretically supported by computations to establish the role of Fermi's golden rule, which states that low frequency vibrational modes play a major contribution in the nonradiative decay of the excited states.^[31]^ Recently, an alternative model, restricted access to a conical intersection (RACI), was introduced by Blancafort and co‐workers in 2013. This model has been implemented to explain the AIE phenomenon exploiting the global potential energy surface (PES).[^32^, ^33^, ^34^, ^35^] Conical intersections (CIs) play a crucial role in photochemical processes, including photochemical reactions and fluorescence quenching, as they facilitate nonadiabatic transitions from excited to ground states.[^36^, ^37^, ^38^] In the RACI model, quenching can effectively compete with fluorescence if the path from the Franck‐Condon (FC) region to a CI is downhill or has a low barrier in solution. However, in the aggregated phase, due to restriction of motion, the nonradiative decay pathways are blocked because the point of conical intersection lies higher in energy than the FC point. The movements that lead to the decay in this mechanism are the rotation around the central C=C bond in diphenyldibenzofulvene (DPDBF)^[32]^ and the twisted shape of the silole ring combined with the flapping of phenyl rings in dimethyl tetraphenylsilole (DMTPS).^[35]^ In the past few years, the RACI mechanism has been proven to be correct for many more compounds that exhibit AIE and the decay process connected to ESIPT, double bond torsion, ring puckering, photocyclization, or bond stretching.^[33]^

Theoretically, the choice of method plays a crucial role in studying the excited state PES. Multiconfigurational approaches,^[39]^ like complete active space self‐consistent field (CASSCF)[^40^, ^41^] consider static correlation effects and are appropriate for treating energy degeneracy at CI. These approaches have some drawbacks, such as they require a defined set of active space orbitals and high computational cost. Moreover, dynamic correlation energy is not included in the CASSCF method. However, it can be complemented with CASPT2[^42^, ^43^] or other comparable methods to account for the latter effect. In such a scenario, time‐dependent density functional theory (TD‐DFT),[^44^, ^45^] which is more computationally efficient and can handle large systems, provides a practical alternative to investigate the PES. This method usually provides a good description of the absorption and emission energies and also includes dynamic correlation. However, it can be challenging to determine the location of a CI with TD‐DFT because of the highly distorted structures near CI where the ground state acquires a multireference character. This, in turn, can cause TD‐DFT to fail. To avoid this problem, the spin‐flip (SF) TD‐DFT method,^[46]^ in which reference wavefunction is an auxiliary triplet state, has been introduced. The SF‐TDDFT method is preferred for locating the CI of medium to large molecules due to its balanced and reliable description of both ground and excited states and its low computational cost.[^47^, ^48^, ^49^] Considering the challenges mentioned earlier, it is expected that the spin‐flip version of TD‐DFT can be a highly effective method for describing the electronic structures of luminogens which exhibit fluorescent quenching in solution but highly fluorescent in aggregate state (i. e., show AIE behavior). This is because SF‐TDDFT can capture double excitation and multireference characters at a similar computational cost to conventional TD‐DFT.

**Figure 1:**
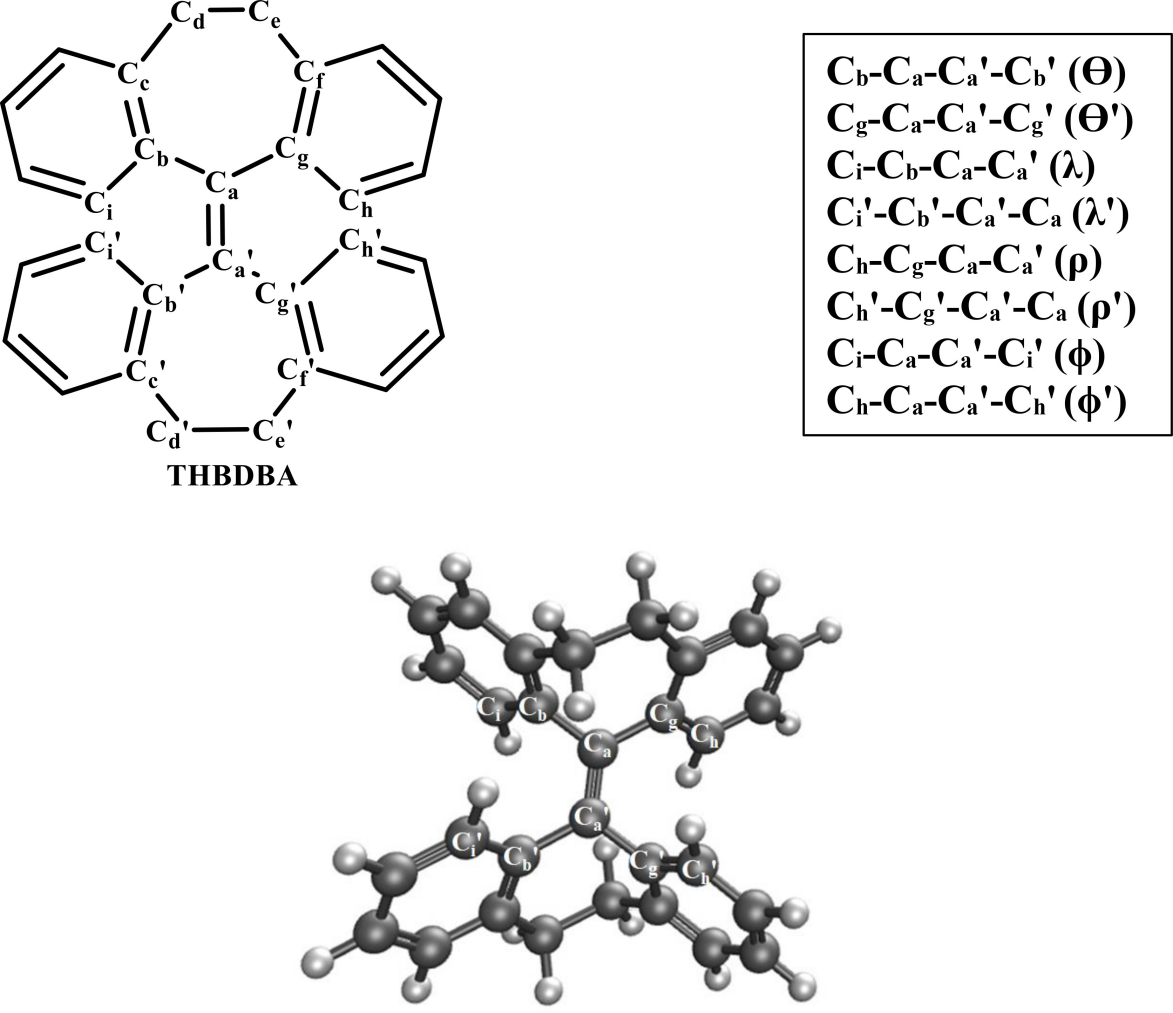
Structure of 10,10’,11,11’‐tetrahydro‐5,5’‐bidibenzo[a,d][7]annulenylidene (THBDBA) and optimized geometry of THBDBA monomer at S_0_ state (S_0_‐MIN) in THF solvent.

In the current study, we aim to expand the RACI model to 10,10′,11,11′‐tetrahydro‐5,5′‐bidibenzo[a,d][7]annulenylidene (THBDBA) that resembles the TPE core structure with covalently linked phenyl rings (see Figure 1), where the RIV model has been used previously to address the AIE phenomenon.^[9]^ THBDBA is a TPE derivative, where its two pairs of phenyl rings are restricted by two singly bonded ethyl chains that are covalently linked, preventing them from rotating freely. In tetrahydrofuran (THF) solution, THBDBA emits significantly less emission (or almost non‐emissive), and the fluorescence quantum yield increases by 230 times in aggregate.

THBDBA had been proposed as a molecular switch, along with other tetrabenzoheptafulvalene derivatives.^[50]^ THBDBA is anti‐conformationally stable and exhibits no anti‐syn isomerization upon irradiation. As a result, it undergoes photocyclization by forming a new C_i_‐C_i_′ bond. This shows that the seven membered rings play an essential role in photoreactivity. The RIV model previously described the AIE behavior of THBDBA using Fermi's golden rule; vibronic coupling simulations revealed that the decrease of vibrational channels and total reorganization energy in the aggregate state generates the AIE of THBDBA, suggesting that in‐plane and out‐of‐plane bending vibrations of phenyl rings determined radiationless decay.^[9]^

The manuscript is structured as follows: A brief theoretical background of the related computational implementation, including a description of RSH functionals, optimization strategy of the monomer and the dimer (i. e., the aggregate state in a minimal scale), etc. In sub‐section 2.1, we first discussed the photophysical properties obtained from RSH functional for both the monomer and the dimer in THF solution. For the monomer system, these values are compared with the experimental ones. Subsequently, in sub‐section 2.2, we have discussed in detail the excited state quenching process in solution using the conical intersection model. To supplement this model, the variation of oscillator strength associated with the corresponding transitions along the PES are also computed. A similar discussion of the dimer system is elaborated in subsection 2.3. The difference in the key parameter (i. e., the minimum energy gap) of the monomer and the dimer systems along the PES is found to be handy in explaining the AIE phenomenon. Finally, in the concluding section (Section 3) we have summarized the whole study as well as the probable approach that needs to be adopted to further narrow down the energy gap between the S_0_ and S_1_ states to consolidate the minimum energy conical intersection (MECI) theory of AIE behavior.

## Computational Methodology

The effectiveness of a method in reproducing intramolecular and intermolecular charge transfer in the excited states is crucial. So choosing suitable functionals in SF‐TDDFT is essential to describe the electronic processes in luminophores accurately. It is important to note that the commonly used hybrid functionals, such as B3LYP, often cannot accurately predict electronic structures due to their inability to handle large amounts of self‐interaction energy (SIE). As a result, the delocalization of electrons is greatly overestimated. The prediction of charge‐transfer excitation energies is the most prevalent domain where linear‐response TDDFT calculations using hybrid functionals are mainly known to fail. The derivative discontinuity and incorrect asymptotic behavior are further reasons for the failure of such functionals.^[51]^ The standard hybrid functionals cannot resolve the aforementioned critical problems in DFT. Therefore, the range‐separated hybrid (RSH) functionals, which eliminates long‐range self‐interaction in the exchange functional,[^52^, ^53^] are necessary to adequately describe charge‐transfer excitations in Donor‐Acceptor type molecules. RSH functionals significantly contribute to the DFT by reproducing multiple characteristics like orbital energies, nonlinear optical properties, Rydberg excitations, etc. Within the framework of RSH functionals, the inter‐electronic coulomb potential is partitioned into short and long‐range components using the error function erf(ωr). These functionals are designed to restore asymptotically corrected 1/r long‐range behavior, thus resulting in accurate charge‐transfer values. The charge‐transfer has been defined by well‐separated and nonoverlapping donor and acceptor orbitals.^[54]^ Therefore, the coulomb attractions can also be obtained between transferred holes and electrons.[^55^, ^56^, ^57^] Choosing the appropriate functional is crucial for accurately describing the electronic states of all components in a single‐molecular system with multiple moieties.

The chemical structure and the optimized geometry of the investigated fluorophore monomer (i. e., the molecule abbreviated as THBDBA) are illustrated in Fig. 1. The dimer system is chosen to mimic the smallest aggregate state. It is constructed by placing two monomers according to the experimental crystallographic data. In the dimer case, only one molecule was allowed to relax during the geometry optimization (lower monomer), while the other molecule was fixed (upper monomer) to create a restricted environment at the smallest level (see Fig. 3c). For both the monomer and the dimer, SF‐TDDFT method was used to calculate the PES at excited state (S_1_) and analytical gradients of the molecule. The minimum energy point of the S_0_ state (S_0_‐MIN) is obtained by geometry optimization. For the S_1_ state, the geometry optimization starts from the FC point to reach the optimized structure (S_1_‐MIN). The electronic structures were generated using RSH functional (i. e., LRC‐ωPBEh),^[58]^ cc‐pVDZ basis set^[59]^ and the solvent effect was addressed via a linear‐response polarizable continuum model (LR‐PCM).[^60^, ^61^] The MECI between the S_0_ and S_1_ states is generated through a potential energy surface (PES) scan at S_1_‐state. To scan the PES, constrained geometry optimization, by fixing the torsional angles φ (C_i_‐C_a_‐C_a_′‐C_i_′), φ′ (C_h_‐C_a_‐C_a_′‐C_h_′), and Θ′ (C_g_‐C_a_‐C_a_′‐C_g_′) (see Figure 1, Table 2), were performed throughout the range of 0⁰‐180⁰ at S_1_ state. Penalty‐constrained optimization method was adopted to determine the conical intersection (CI) hypersurface between the S_0_ and S_1_ states (i. e., S_0_/S_1_ MECI).[^62^, ^63^] The constrained geometry optimization of torsional angles φ, φ′, and Θ′ results in CI of S_0_/S_1_ MECI, S_0_/S_1_ MECI′, and S_0_/S_1_ MECI′′, respectively. All the electronic structure calculations mentioned above were performed using the quantum chemistry software Q‐Chem 5.3.^[64]^

## 2 Results and Discussion

As pointed out in the Introductory Section (paragraph 5, page 2), in 2011,^[50]^ Luo et al. synthesized a series of overcrowded tetrabenzoheptafulvalene derivatives, promising building blocks for molecular switches and machines. In 2014,^[9]^ Leung et al. used one of the reported molecule by Luo et al^[50]^ and reported it to be 10,10’,11,11’‐tetrahydro‐5,5’‐bidibenzo[a,d][7]annulenylidene (THBDBA). They found THBDBA is significantly less emissive in the solution phase but is highly emissive in the crystalline phase i. e., AIE active. The computational study, using the QM/MM model, revealed that the mechanism behind the AIE activity of THBDBA was the RIV of phenyl rings.^[9]^ The theoretical tool they used is general Amber force field (GAFF) for MM part (116 surrounding molecules) and B3LYP/6‐31G* for the QM part (one central molecule). In 2019, Ding et al. reported a fully quantum mechanical (QM) study of the same molecule using MS‐CASPT2//CASSCF level and OM2/MRCI nonadiabatic dynamics simulations.^[65]^ They observed the existence of S_0_/S_1_ CI in solution. Without explicitly carrying out any quantum mechanical computation they argued in favor of the role of RACI for the AIE properties of THBDBA in aggregate phase. Blocking of large displacements (required for non‐radiative photocyclization process that leads to conical intersection) in aggregate phase was conjectured to be the cause of AIE behavior.

In the current work, we aim to analyze the photo‐physics and photo‐chemistry of THBDBA in order to assess the reliability of the RACI model in investigating its AIE behavior. To accomplish this objective, we performed calculations to map out the PES of the molecule in its excited state to provide a deeper understanding of nonradiative decay at the molecular level. It is expected that the use of SF‐TDDFT method, which is computationally effective, will be helpful to show that RACI is a more fundamental cause of the photoluminescence properties and thus re‐confirm the success of this model in explaining AIE phenomena. All the outcomes are addressed in several subsections as indicated below:

### 2.1 Electronic Structures and Optical Spectrum

The picture of the THBDBA molecule is shown in Figure 1. Using SF‐TDDFT, the S_0_ and S_1_ states are optimized with the LRC‐ωPBEh functional, cc‐pVDZ basis set, and LR‐PCM handles the Solvent effects. Table 1 lists the computed vertical excitation and de‐excitation energies at S_0_‐MIN and S_1_‐MIN, i. e., the theoretical absorption (S_0_→S_1_) and emission energies (S_1_→S_0_). The corresponding experimental values^[9]^ are also reported. From the computed values we can conclude that for monomer in THF solvent, the longest vertical transition (i. e., S_0_→S_1_) wavelength (i. e., theoretical absorption wavelength) is 266 nm (4.66 eV) with the highest oscillator strength (*f*) value as 0.6919 a.u. This value is in quite good agreement with the experimentally obtained absorption wavelength (273 nm). Furthermore, as per the experimental study, the molecule is non‐emissive in solution. It is quite encouraging to note that the theoretically calculated oscillator strength (*f*) value of the corresponding emission transition (i. e., S_1_→S_0_) aptly supports the experimental observations. The negligibly small *f*‐value (i. e., 0.0038 a.u.) perfectly aligns with the quenching phenomena as claimed by the experimental study. For the dimer system, no experimental data are available to compare with the theoretically computed ones. This is because the dimer system is a hypothetical one, chosen to mimic the aggregate state in a miniature form. From Table 1 it is obvious that in the THF solvent there is a slight increment in the calculated absorption wavelength (269 nm) when compared to that of the monomer, which seems reasonable. The computed emission wavelength for S_1_→S_0_ is found to be 376 nm with *f*=0.5641 a.u. This value is quite close to the experimental emission wavelength in the aggregate state (380 nm).^[9]^ In addition, the commonly observed spin‐contamination problem related to the spin‐flip approach is not profound in this study, as indicated by the $\left\langle S^{2}\right\rangle$ values of states S_0_ and S_1_ listed in Table S1.

**Table 1:** Theoretically generated absorption (S_0_→S_1_) and emission (S_1_→S_0_) wavelengths (in nm) for both the monomer and the dimer of the THBDBA molecule in THF solvent. The method used is SF‐TDDFT/LRC‐ωPBEh/cc‐pVDZ/THF. The corresponding experimental values (whichever are available) are also listed.

System*	Vertical Abs. Transition	OscillatorStrength (S_0_→S_1_) (*f*) (a.u.)	Cal. Abs. (nm)	*Exp. Abs. (nm)	Oscillator Strength (S_1_→S_0_) (*f*) (a.u.)	Cal. Emi. (nm)	*Exp. Emi. (nm)
THBDBA (Monomer in THF)	S_0_→S_1_	0.6919	266	273	0.0038 (non‐emissive)	–	Not available
THBDBA (Dimer in THF)	S_0_→S_1_	0.6946	269	–	0.5641	376	380 (Agg.)

*^[9]^

### 2.2 Quenching of Fluorescence/ Radiation‐Less Decay Pathway of Monomer in THF Solution

For the THBDBA monomer and dimer, all the optimized geometrical parameters, such as bond lengths and dihedral angles, are listed in Table 2. The optimized structure at S_0_‐MIN is found to have anti‐ conformation as depicted in Figure 1. Interestingly, this also what is observed experimentally.^[50]^

**Table 2:** Relevant geometrical parameters (as per Figure 1) of critical points, such as, S_0_‐MIN, S_1_‐MIN, S_1_/S_0_‐MECI (restricted motion around φ), S_1_/S_0_‐MECI′ (restricted motion around φ′), and S_1_/S_0_‐MECI′′ (restricted rotation around Θ′), of THBDBA (for both monomer and dimer) in solution obtained from computational calculations. The method used is SF‐TDDFT/LRC‐ωPBEh/cc‐pVDZ/LR‐PCM, THF.

	Monomer					Dimer	
Parameters Dihedral (⁰), Bond length (Å)	S_0_‐MIN	S_1_‐MIN	S_1_/S_0_‐MECI	S_1_/S_0_‐MECI′	S_1_/S_0_‐MECI′′	S_0_‐MIN	S_1_‐MIN (Local‐MIN)
C_b_‐C_a_‐C_a_′‐C_b_′ (Θ)	−6.3	−3.9	53.2	−4.3	72.7	−5.8	−15.4
C_g_‐C_a_‐C_a_′‐C_g_′ (Θ′)	6.3	21.4	38.3	21.8	105.3	6.6	16.5
C_i_‐C_b_‐C_a_‐C_a_′ (λ)	−62.4	−30.8	−47.8	−30.8	27.6	−62.3	−44.1
C_i_′‐C_b_′‐C_a_′‐C_a_ (λ′)	−49.4	−23.1	50.0	−13.8	−37.2	−48.4	−33.8
C_h_‐C_g_‐C_a_‐C_a_′ (ρ)	49.4	38.9	13.5	38.1	−46.4	49.3	33.5
C_h_′‐C_g_′‐C_a_′‐C_a_ (ρ′)	62.4	50.3	−14.1	48.8	22.7	61.6	43.4
C_i_‐C_a_‐C_a_′‐C_i_′ (φ)	−55.5	−30.9	56.0	−26.8	68.4	−54.5	−52.3
C_h_‐C_a_‐C_a_′‐C_h_′ (φ′)	55.5	63.3	38.4	62.8	96.2	55.6	53.1
C_a_‐C_a_′	1.34	1.43	1.45	1.44	1.43	1.34	1.45
C_i_‐C_i_′	3.39	2.10	3.18	1.81	3.29	3.35	2.94
C_h_‐C_h_′	3.39	3.26	1.99	3.22	3.70	3.38	2.95

**Figure 2:**
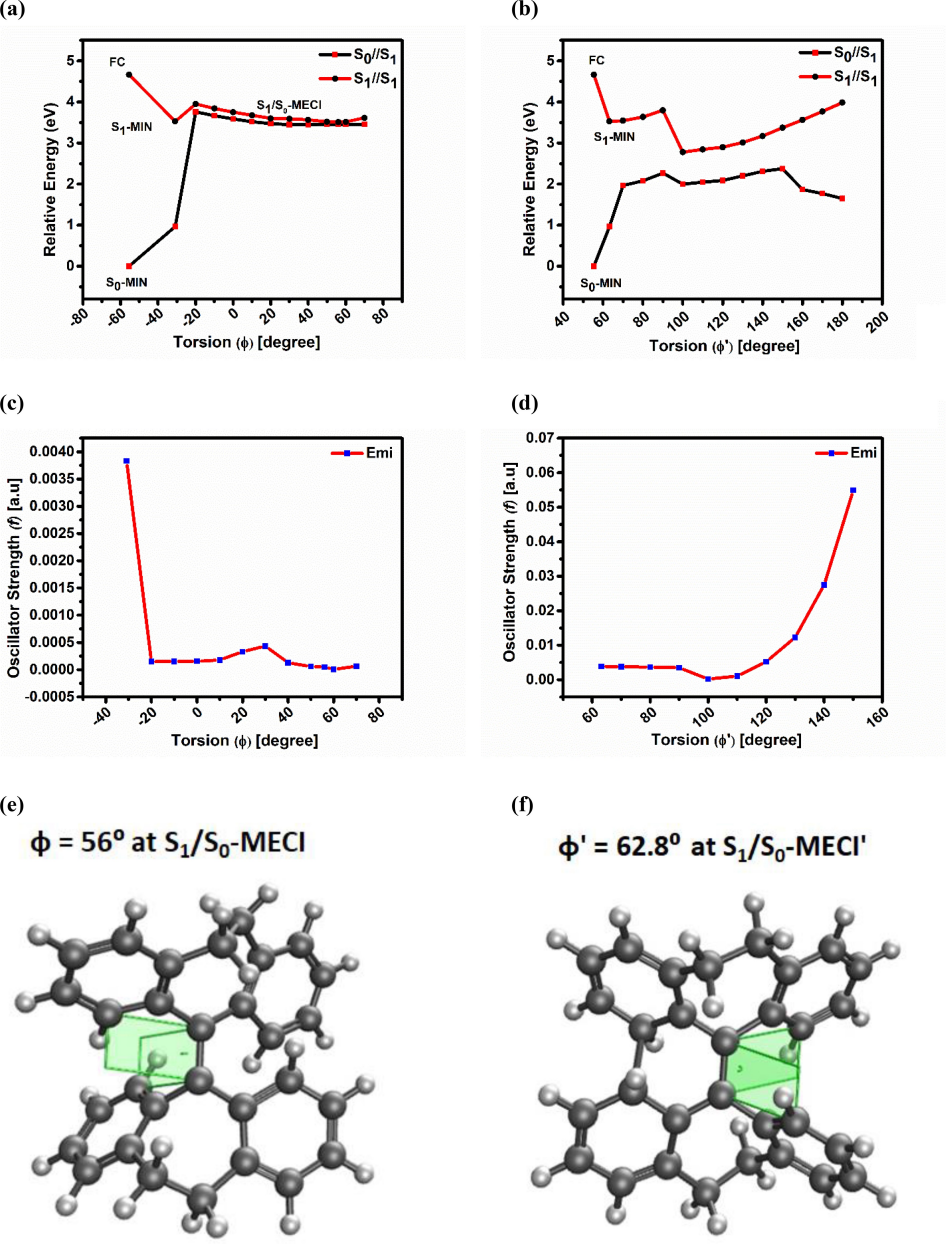
(a) Plot of potential energy profile along S_1_‐state i. e., S_1_‐MEP generated from restricted PES scan around φ torsion angle in THF solution (calculated with SF‐TDDFT/LRC‐ωPBEh/cc‐pVDZ/LR‐PCM, THF) (b) Plot of potential energy profile along S_1_‐state i. e., S_1_‐MEP around φ′ torsion angle in THF solution (calculated with SF‐TDDFT/LRC‐ωPBEh/cc‐pVDZ/LR‐PCM, THF) (c) Plot of oscillator strength value vs torsion angle (φ) along S_1_‐MEP (d) Plot of oscillator strength values vs torsion angle (φ′) along S_1_‐MEP (e) Optimized geometry corresponding to S_1_/S_0_‐MECI (φ=56⁰) along S_1_‐MEP (f) Optimized geometry corresponding to S_1_/S_0_‐MECI′ (φ′=62.8⁰) [Using penalty constrained optimization algorithm along S_1_‐MEP].

The significant change induced by the excitation from S_0_‐MIN to S_1_‐MIN induces an inversion of the bond pattern within the molecule. Specifically, the central C_a_‐C_a_′ bond length increases from 1.34 Å to 1.43 Å, while the distance between the C_i_ and C_i_′ atoms decreases from 3.39 Å to 2.10 Å. This indicates that the phenyl rings, including the C_i_ and C_i_′ atoms, are moving closer together, forming a new C_i_‐C_i_′ bond. As shown in Figure 2f, the C_i_‐C_i_′ distance decreases to 1.81 Å at the S_1_/S_0_‐MECI’ and forms a new benzene ring (C_a_‐C_b_‐C_i_‐C_i_’‐C_b_’‐C_a_’) that leads to the ring closed product. A detailed analysis is provided in section 2.2.2. Additionally, Table 2 demonstrates significant changes in torsional angles. We specifically focus on the torsional angles that facilitate the flapping motion of the phenyl rings (i. e., φ and φ’) as well as those associated with anti‐syn isomerization (i. e., Θ and Θ′). Upon excitation from S_0_‐MIN to S_1_‐MIN, the φ torsion angle shifts from −55.5⁰ to −30.9⁰. Meanwhile, the λ and λ′ torsion angles decrease by 31.6⁰ and 26.3⁰, respectively, confirming the presence of intramolecular vibrations in the solution phase. Regarding anti‐syn isomerization, the Θ′ torsion angle increases from 6.3⁰ to 21.4⁰, indicating notable rotation around the C_a_‐C_a_′ bond. We have thoroughly calculated the PES for both torsional angles (φ and Θ′). The torsion angles Θ′, φ, and φ’ are measured at 105.3⁰, 68.4⁰, and 96.2⁰, respectively, at the S_1_/S_0_‐MECI’’, indicating that this structure is inherently challenging to access. The non‐radiative decay at the molecular level is clearly a result of vibrational motion over anti‐syn isomerization in the THBDBA molecule. Additionally, comprehensive analyses of the PES scans for the angles φ and Θ′ are thoroughly presented in sections 2.2.1 and 2.2.3, respectively.

#### 2.2.1 Restricted Motion Around φ Torsion of a Monomer in THF Lead to S_1_/S_0_‐MECI

To further explore the pathway of fluorescence quenching (or radiation‐less decay) of the molecule the PES at the excited state is scanned. As discussed in the previous paragraph (as well as obvious from the data of Table 2) in the solution the predominant distortion, i. e., the flapping motion of phenyl rings, is observed from S_0_‐MIN→S_1_‐MIN, which corresponds more to λ, λ′ as well as φ torsion angles, in comparison to ρ, ρ′, and φ′. Based on these observations, we have performed the PES scan via the φ torsion angle. Figure 2a illustrates the PE profile of S_1_‐Minimum Energy Pathway (MEP) after conducting constrained geometry optimization of the φ torsion angle with a step of 10⁰ in the solution. This PES scan is done to examine the impact of flapping motion of the phenyl rings on the degeneracy of S_0_ and S_1_ states. As tabulated in Table 3, along the S_1_‐MEP, the energy of S_1_‐MIN is 3.52 eV at φ=‐30.9⁰, which is 1.14 eV lower than the FC point (4.66 eV). The scanned S_1_‐state energy barrier is 0.43 eV higher than S_1_‐MIN and 0.71 eV lower than the FC point at φ=‐20⁰ (given in Table S2), suggesting that it is easily accessible to reach the S_1_/S_0_‐MECI. At φ=56⁰ along the S_1_‐MEP the energy values of the S_1_ and S_0_ states are 3.51 eV and 3.46 eV, respectively, (i. e., a difference of 0.05 eV). Thus, at this point the S_1_ and S_0_ states can be considered as almost degenerate and so S_1_/S_0_‐MECI (as shown in Table 3 and Figure 2a, 2e). The relative energy value of the S_1_ state at S_1_/S_0_‐MECI is 3.51 eV, i. e., almost similar to S_1_‐MIN (i. e., 3.52 eV).

**Table 3:** Relative energies (in eV) of the Key points of THBDBA (for monomer and dimer) in solution obtained from computational calculations (SF‐TDDFT/LRC‐ωPBEh/cc‐pVDZ/LR‐PCM, THF).

System	Geometry	S_0_‐State	S_1_‐State
Monomer	S_0_‐MIN	0.00	4.66
	S_1_‐MIN	0.97	3.52
	S_1_/S_0_‐MECI	3.46	3.51
	S_1_/S_0_‐MECI′	3.49	3.62
	S_1_/S_0_‐MECI′′	3.05	3.17
Dimer	S_0_‐MIN	0.00	4.64
	S_1_ (Local‐MIN)	0.66	3.96

So, it can be concluded that in the pathway from FC point to S_1_/S_0_‐MECI, the excited state molecule returns to the S_0_‐MIN through non‐radiative decay and exhibits fluorescence quenching. Additional evidence that supports the non‐radiative decay pathway of THBDBA in solution is the change in oscillator strength. As tabulated in Table 1, the theoretically calculated *f*‐value of the emission transition (S_1_→S_0_) (at S_1_‐MIN) is 0.0038 a.u., justifying the experimental observation of quenching in solution. To confirm this, we have plotted the *f*‐values along S_1_‐MEP (as depicted in Figure 2c), and all the corresponding values are detailed in Table S3. It is evident that the *f*‐values throughout the PES scan tends to be close to zero, and at S_1_/S_0_‐MECI (φ=56⁰), *f*=0.00005 a.u., validating almost total fluorescence quenching.

#### 2.2.2 Restricted Motion Around φ′ Torsion of a Monomer in THF Lead to S_1_/S_0_‐MECI′

So far we have demonstrated the S_1_/S_0_‐MECI based on the φ torsion swing motion. However, another critical factor also indicates a change is the inversion of the bond pattern. Specifically, the distance between C_i_‐C_i_′ is shortened from 3.39 Å to 2.10 Å during the excitation from S_0_‐MIN to S_1_‐MIN in solution (Table 2). In 2011,^[50]^ Luo and co‐workers experimentally confirmed the formation of ring‐closed product during photo‐cyclization. In 2019,^[63]^ Ding and co‐workers theoretically showed, using the MS‐CASPT2//CASSCF method, that the decay path from S_1_ led to a CI and then to S_0_ via a cyclization process involving two neighboring phenyl rings with a gap of 0.26 eV at S_1_/S_0_‐MECI. Based on this observation, we have chosen one more torsion angle φ′ for the PES scan (Figure 2b) to observe the change in distance between two neighboring phenyl rings (bond C_i_‐C_i_′) and led to ring‐closed product (C_a_‐C_a_′‐C_b_′‐C_i_′‐C_i_‐C_b_) at MECI. As the φ′ torsion angle increases, the S_1_‐state energy profile gradually decreases after an uphill of 0.27 eV at φ′=90⁰ and reaches a MEG structure of 0.78 eV at φ′=100⁰ (listed in Table S5).

Although the PES of the S_0_ and S_1_ states fail to demonstrate the phenomenon of CI, the existence of the same can be established through alternative evidence. The alternative evidence is the expected change in oscillator strength (*f*) throughout the PES and near the CI region (shown in Figure 2d). Table S6 lists the corresponding values. It is observed that the value of *f* at S_1_‐MEP tends to be zero within the range of φ′=63⁰ to 120⁰, which is also an evidence of the non‐radiative internal conversion (or access to conical intersection), resulting in fluorescence quenching. In order to reach the MECI, we further conducted penalty‐constrained optimization of the S_1_ state using the SF‐TDDFT method. We reached the S_0_/S_1_‐MECI′ (3.56 eV) with a gap of 0.13 eV at φ′=62.89⁰ (listed in Table 4), which is narrower than the previously reported value^[63]^ and lies 1.10 eV lower than FC point (4.66 eV). Figure 2f depicts the optimized geometry at S_1_/S_0_‐MECI′ of the molecule THBDBA. The *f*‐values at this S_1_/S_0_‐MECI′ reach as low as 0.0005 a.u. (Table 4), indicating that excited state energies are dissipated almost entirely through a non‐radiative decay pathway. We also identified a ring‐closed product at S_1_/S_0_‐MECI′ (Figure 2f). As a result, in both cases (i. e., S_1_/S_0_‐MECI and S_1_/S_0_‐MECI′), the non‐radiative energetic decay at the intersection is feasible in the solution, which leads to fluorescence quenching.

**Table 4:** Calculated parameters at Minimum Energy Conical Intersection (MECI) using the Penalty Constrained Search algorithm, as available in Q‐Chem 5.3.^[62]^

System*	torsion (⁰)	State	E.S. Energy at MECI (eV)	Energy at MECI (E_MECI_) (eV)*	Energy diff. at MECI (eV)	*f* (a.u.) (S_1_ →S_0_)
Monomer	(φ′) 62.8	E.S‐1 (S_0_) E.S‐3 (S_1_)	3.49 3.62	3.56	0.13	0.0005
	(Θ′) 105.3	E.S‐2 (S_0_) E.S‐3 (S_1_)	3.05 3.17	3.11	0.12	0.0007

* optimized using SF‐TDDFT/LRC‐ωPBEh/cc‐pVDZ/THF, energy values at Column 5^th^ is the average energy of the S_0_ and S_1_ states from column 4^th^.

#### 2.2.3 Restricted Rotation Around Θ′ Torsion of a Monomer in THF Lead to S_1_/S_0_‐MECI′′

We further discovered a MECI for anti‐syn isomerization, S_1_/S_0_‐MECI′′, that incorporates a twisted central bond, which helps us better comprehend the switching pattern of THBDBA. The torsion angles for the twisted central bond, denoted as Θ and Θ′, have values of 72.7⁰ and 105.3⁰, respectively at S_1_/S_0_‐MECI′′. Using the penalty‐constrained optimization algorithm, we were able to reach the S_1_/S_0_‐MECI′′, which is energetically 1.55 eV lower than the FC point. The relative energy value of the S_1_ state at S_1_/S_0_‐MECI′′ is 3.17 eV, i. e., lower than S_1_‐MIN by 0.35 eV. Figure S2 shows the orbitals involved in the excitation at the S_1_/S_0_‐MECI′′. The position of the S_1_ state orbitals at the S_1_/S_0_‐MECI′′ differs from those at S_1_‐MIN. At the S_1_/S_0_‐MECI′′ structure, we observe modest pyramidalization (almost 23⁰) at one of the carbon atoms (Figure S1). This differs from similar structures encountered in methylated TPE derivatives, which display a more significant pyramidalization (almost 43⁰) and a twist around the central double bond and have been linked to E/Z isomerization.^[66]^ All the MECI structures, namely S_1_/S_0_‐MECI, S_1_/S_0_‐MECI′, and S_1_/S_0_‐MECI′′, are energetically lower than the FC structure by approximately 1.15 eV, 1.04 eV, and 1.49 eV, respectively. However, to reach the S_1_/S_0_‐MECI′′ structure, significant steric repulsion need to overcome between C_i_ and C_i_′, and C_h_ and C_h_′. This suggests that this structure will not be easily accessible compared to S_1_/S_0_‐MECI and S_1_/S_0_‐MECI′.

### 2.3 Radiative Decay Pathway of Dimer in THF Solution

**Figure 3:**
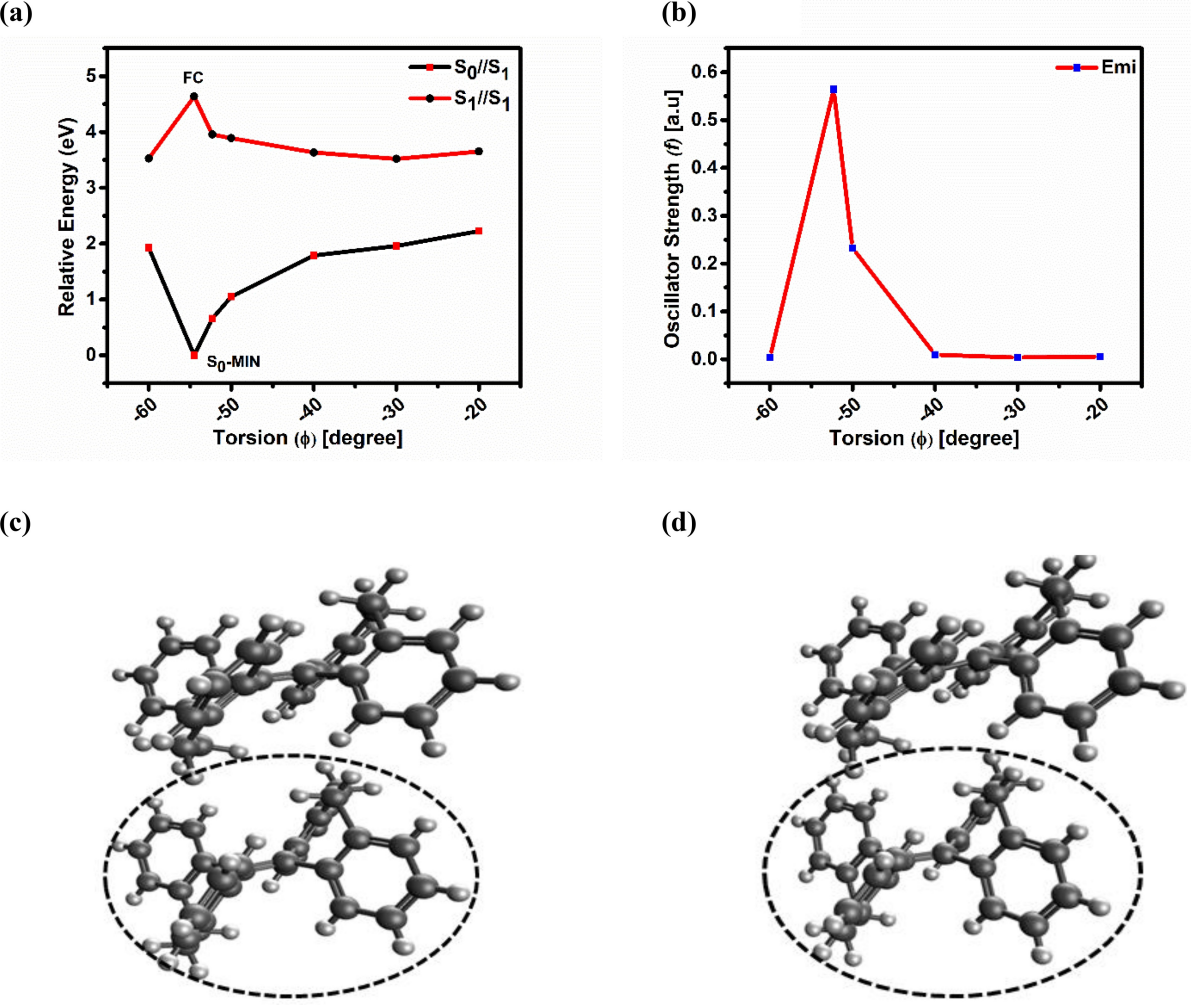
(a) Plot of potential energy profile along S_1_‐state i. e., S_1_‐MEP of dimer around φ torsion angle in THF solution (calculated with SF‐TDDFT/LRC‐ωPBEh/cc‐pVDZ/LR‐PCM, THF) (b) Plot of oscillator strength values vs torsion angle (φ) along S_1_‐MEP (c) Optimized S_0_‐state geometry (S_0_‐MIN) (d) Optimized S_1_‐state geometry (S_1_‐Local‐MIN), In (c) and (d) the circled monomer (i. e., the lower one) is the relaxing one and the upper one is fixed during the geometry optimization at ground and excited states.

To mimic the aggregate state, we have taken the smallest unit, a dimer, and performed the electronic structure calculations on this system in THF using the SF‐TDDFT method. In our current study, we also found that for the dimer system, the changes in the geometrical parameters of S_0_ and S_1_ optimized states were restricted, compared to the monomer system, due to some steric and electrostatic confinements. From the geometrical parameters in Table 2, we can see that the changes in φ and φ′ torsion angles in the dimer are restricted to 2.2⁰ and 2.5⁰, respectively, when the system moves from S_0_‐MIN to S_1_‐MIN. The corresponding changes in the φ and φ′ torsion angles in the monomer are 24.6⁰ and 7.8⁰, respectively. So, it is quite logical to argue that the φ and φ′ torsion angles in the dimer are more restricted when compared to the monomer. In monomer, another significant change induced by the excitation from S_0_‐MIN→S_1_‐MIN was the shortening of the distance between C_i_‐C_i_′ by 1.29 Å. In contrast, in the dimer, the distance between C_i_‐C_i_′ is only decreased by 0.41 Å (see values from Table 2). Additionally, as shown in Figures 3c and 3d, it is evident that the two monomers are stacked on top of each other. There seems to be a limitation on the flapping motion of the relaxing molecule due to steric hindrance from the surrounding fixed molecule. The energy difference between the FC point and the S_1_‐MIN for the monomer in solution is 1.14 eV. In contrast, the change in energy for the dimer is 0.68 eV, indicating that less excessive energy generates a small driving force for intramolecular motions in the dimer.

Due to the flapping motion of the φ and φ′ torsion, the conical intersection lies below the FC point in the monomer case. To prove that in the aggregated state nonradiative decay pathways are inhibited we need to show that the conical intersection is energetically higher than the FC point. So, a PES scan via the φ torsion angle of the dimer system was conducted. Starting from the FC structure, constrained geometry optimization at S_1_ state is carried out, fixing the torsional angle φ (see Figure 3a). A comparison between Figures 2a and 3a clearly shows that the PES of the dimer system significantly differs from that of the THBDBA monomer in THF. At a dihedral angle (φ) of −20⁰, the energy difference between the S_0_ and S_1_ states of the dimer system is 1.29 eV more than that of the monomer (Table S11). As we move to the right end, the gap between the two states will likely to be larger than the monomer due to some restriction in the molecule caused by some steric and electrostatic confinements, making it unnecessary to compute the S_1_/S_0_‐MECI. As tabulated in Table 1, the theoretically calculated *f*‐value is 0.5641 a.u. for the emission transition (S_1_→S_0_) indicating that when molecules start aggregating, the *f*‐value should also increase leading to intense emission. This argument is also consistent with the experimental observation. To verify this, we examined the *f*‐value at S_1_‐MEP (as depicted in Figure 3b), and all the corresponding values are detailed in Table S12. However, there is only little increase in the *f*‐values of the dimer than that of the monomer.

Unexpected low *f*‐values at the MEG structure in the dimer system is intriguing and needs some exploration. One explanation could be that in the dimer only two monomer units are placed on top of each other, leaving the surrounding sides empty and free of any additional monomers (which is not the situation in the actual crystal). As a result, during the constrained PES scanning the rotating monomer repositioning itself to accommodate the steric and electrostatic repulsion caused by the fixed monomer, behaving almost like an independent monomer. This causes the dimer system more stabilized which produces less pronounced outcomes. However, when more monomer units are present in the surrounding environment, the constraints on motion increase significantly, leading to more expected results (i. e., larger S_1_ – S_0_ energy difference and higher oscillator strength values at the MEG structure). So, the nonradiative decay pathways will be significantly obstructed in the aggregate phase. Consequently, the point of conical intersection is anticipated to be at a higher energy than the Franck‐Condon point, resulting in radiative decay.

## 3 Conclusions

In the present study, the origin of AIE phenomena, demonstrated by the photoluminescent molecule THBDBA, is explored through electronic structure methods. To locate the MEG structure the PES of the S_0_ and S_1_ states of the monomer at S_1_‐optimized geometry are generated by the flapping motion of the phenyl rings (i. e., torsion angle φ and φ′). The relative energy values of the S_0_ and S_1_ states at S_1_/S_0_‐MECI (φ torsion angle) are 3.46 eV and 3.51 eV, respectively. The *f*‐value at this almost degenerate (with a difference of 0.05 eV) geometry (at φ=56⁰) is close to zero (i. e., *f*=0.00005), indicating an almost total fluorescence quenching. However, for the φ′ torsion angle using the penalty‐constrained optimization method at the SF‐TDDFT level we reached the S_1_/S_0_‐MECI′ when the potential energies of the S_0_ and S_1_ states were almost degenerate (within 0.13 eV) at φ′=62.8⁰. Moreover, the drastic decrease of the *f*‐value (S_1_ →S_0_) at S_1_/S_0_‐MECI′ (*f=*0.0005 a.u.) justifies the nonradiative decay of the excited state energy and hence fluorescence quenching.

A thorough analysis of the potential energy profile of the dimer reveals that the flappings of the phenyl rings are restricted when compared to that of the monomer. The dimer also exhibits a notable difference in the S_0_ and S_1_ PESs and a slight rise in the *f*‐values compared to that of the monomer. Because dimer arrangement leads to fewer constraints, the rotating monomer can relax during the restricted PES scanning, leading to less noticeable changes (from that of the monomer) in potential energy difference (between S_0_ and S_1_ states) and *f*‐values at the MEG structure. It is expected that increasing the size of the system from a dimer to an aggregate state would significantly block the non‐radiative decay pathways (due to enhanced steric and electrostatic confinements of the chosen monomer from all directions) and thus substantially enhancing the *f*‐values. As a result, the point of conical intersection is expected to lie higher in energy than the FC point, leading to radiative decay and intense fluorescence.

With most of the aspects taken into account, the methodology employed in the present study combines electronic structure computations to replicate relevant photophysical characteristics and investigate possible explanations for AIE in the aggregate state of the THBDBA molecule. This strategy can be implemented to develop novel photochemical compounds rationally. All in all, the approach has potential utility in the field of photochemistry and related applications.

## Conflict of Interests

The authors declare no conflict of interest.

4

## Supporting information

As a service to our authors and readers, this journal provides supporting information supplied by the authors. Such materials are peer reviewed and may be re‐organized for online delivery, but are not copy‐edited or typeset. Technical support issues arising from supporting information (other than missing files) should be addressed to the authors.

Supporting Information

## Data Availability

The data that support the findings of this study are available in the supplementary material of this article.
