# Supplementary material for: Investigation of Aggregation Induced Emission Mechanism of Tetrabenzoheptafulvalene Derivative by Spin‐Flip Time‐Dependent Density Functional Theory (SF‐TDDFT)
Source: Chem Asian J. 2025 Feb 26;20(9):e202401617. doi: 10.1002/asia.202401617 (PMC12067866; doi:10.1002/asia.202401617)
Supplement: Supplementary file 1 — Supporting Information [file ASIA-20-e202401617-s001.pdf]

Chemistry - An Asian Journal

Supporting Information

Investigation of Aggregation Induced Emission Mechanism of Tetrabenzoheptafulvalene Derivative by Spin-Flip Time-Dependent Density Functional Theory (SF-TDDFT)

Aarzoo and Ram Kinkar Roy*

Investigation of Nonradiative Relaxation of Tetrabenzoheptafulvalene Derivative by Spin-Flip Time Dependent Density Functional Theory (SF-TDDFT): A Computational Approach to Explore the Aggregation-Induced Emission Mechanism

Aarzoo, Ram Kinkar Roy*

AFFILIATIONS:

Dept. of Chemistry, BITS-PILANI, Pilani Campus, Rajasthan, India

***Author to whom correspondence should be addressed:** rkroy@pilani.bits-pilani.ac.in

Supporting Information

	Contents	Page
Table S1	$\langle S^2 \rangle$ values for S_0 and S_1 at S_0 and S_1 optimized geometries	3
Table S2	Potential energy difference between S_0 and S_1 states vs torsional angle ϕ at S_1-MEP for monomer in THF solution	3
Table S3	Oscillator strength *(f)* values (ϕ) at S_1 -MEP of monomer in THF solution	4
Table S4	$\langle S^2 \rangle$ values for S_0, T_1, S_1 states at S_1-MEP along ϕ of monomer in THF solution.	4
Table S5	Potential energy difference between S_0 and S_1 states vs torsional angle ϕ' at S_1-MEP for monomer in THF solution	7
Table S6	Oscillator strength *(f)* values (ϕ') at S_1 -MEP of monomer in THF solution	8
Table S7	$\langle S^2 \rangle$ values for S_0, T_1, S_1 states at S_1-MEP along ϕ' of monomer in THF solution.	9
Table S8	Potential energy difference between S_0 and S_1 states vs torsional angle ϴ' at S_1-MEP for monomer in THF solution	11
Table S9	Oscillator strength *(f)* values (ϴ') at S_1 -MEP of monomer in THF solution	12
Table S10	$\langle S^2 \rangle$ values for S_0, T_1, S_1 states at S_1-MEP along ϴ' of	13

	**monomer in THF solution.**	
**Figure S1**	**(a) Plot of potential energy profile along S_1-state i.e., S_1-MEP using ϴ' torsion angle in THF solution (b) Plot of oscillator strength value vs torsion angle (ϴ') along S_1-MEP (c) Optimized geometry corresponding to S_1/S_0-MECI'' (ϴ' = 105.3^0)**	**16**
**Figure S2**	**Molecular orbitals of THBDBA monomer at the S_0-MIN, S_1-MIN, S_1/S_0-MECI, S_1/S_0-MECI', and S_1/S_0-MECI''**	**16**
**Table S11**	**Potential energy difference between S_0 and S_1 states vs torsional angle ϕ at S_1-MEP for dimer in THF solution**	**21**
**Table S12**	**Oscillator strength *(f)* values (ϕ) at S_1 -MEP of dimer in THF solution**	**21**
**Table S13**	**$\langle S^2 \rangle$ values for S_0, T_1, S_1 states at S_1-MEP along ϕ of dimer in THF solution.**	**22**
**S1**	**Cartesian coordinates of S_0-MIN, S_1-MIN, S_1/S_0-MECI, S_1/S_0-MECI', and S_1/S_0-MECI'' for THBDBA monomer in THF**	**23**
**S2**	**Cartesian coordinates of S_0-MIN, S_1-MIN for THBDBA dimer in THF**	**30**

Table S1. $\langle S^2 \rangle$ **values for S_0 and S_1 at S_0 and S_1 optimized geometries.**

Molecule (THBDBA)	$\langle S^2 \rangle_{S_0}$ at S_0 Opt.	$\langle S^2 \rangle_{S_1}$ at S_0 Opt.	$\langle S^2 \rangle_{S_0}$ at S_1 Opt.	$\langle S^2 \rangle_{S_1}$ at S_1 Opt.
**Monomer**	0.0516	0.2550	0.0391	0.0395
**Dimer**	0.0526	0.2506	0.0842	0.1957

Table S2. Tabulated potential energy difference between S_0 and S_1 states vs torsional angle ϕ at S_1-MEP for monomer in THF solution.

Serial No.	Dihedral Angle (ϕ) value (in degrees)	S_0-state Energy (in eV)	S_1-state Energy (in eV)	Difference in Energy (in eV)
**1**	**-55.5**	0 (S_0-MIN)	4.665 (FC)	4.665
**2**	**-30.9**	0.97	3.527 (S_1-MIN)	2.557
**3**	**-20**	3.760	3.953	0.193
**4**	**-10**	3.667	3.843	0.176
**5**	**0**	3.587	3.752	0.165
**6**	**10**	3.518	3.676	0.158
**7**	**20**	3.472	3.602	0.130
**8**	**30**	3.447	3.591	0.144
**9**	**40**	3.451	3.566	0.115
**10**	**50**	3.459	3.519	0.060
**11**	**56**	3.456	3.510	0.054
**12**	**60**	3.458	3.515	0.057
**13**	**70**	3.457	3.615	0.158

Table S3. Tabulated oscillator strength *(f)* values along ϕ torsional angle at S_1 -MEP of monomer in THF solution.

Serial No.	Dihedral Angle (ϕ) value (in degrees)	Oscillator Strength (*f*) (in a.u.) ($S_1 \rightarrow S_0$)
**1**	**-30.9 (S_1-MIN)**	0.003833
**2**	**-20**	0.000149
**3**	**-10**	0.000152
**4**	**0**	0.000155
**5**	**10**	0.000178
**6**	**20**	0.000332
**7**	**30**	0.000433
**8**	**40**	0.000130
**9**	**50**	0.000059
**10**	**56**	0.000058
**11**	**60**	0.000007
**12**	**70**	0.000064

Table S4. $\langle S^2 \rangle$ values for Excited state 1, Excited state 2 and Excited state 3 along ϕ torsional angle at S_1-MEP of monomer in THF solution.

Serial No.	Dihedral Angle (ϕ) (in degrees)	S_1 PES (States)	$\langle S^2 \rangle$ Values
**1**	**-55.5 (FC)**	1 (might be the ground state)	0.0516
		2	2.0326
		3	0.2550
		4	1.0529
**2**	**-30.9 (S_1-MIN)**	1 (might be the ground state)	0.0391

		2	2.0283
		3	0.0395
		4	1.0537
**3**	**-20**	1 (might be the ground state)	0.5237
		2	1.6077
		3	0.4457
		4	0.2909
**4**	**-10**	1 (might be the ground state)	0.5175
		2	1.4812
		3	0.5808
		4	0.2933
**5**	**0**	1 (might be the ground state)	0.4986
		2	1.3300
		3	0.7430
		4	0.2909
**6**	**10**	1 (might be the ground state)	0.4705
		2	1.1343
		3	0.9379
		4	0.2779
**7**	**20**	1 (might be the ground state)	0.4138
		2	0.8460
		3	1.2161
		4	0.2518
**8**	**30**	1 (might be the ground state)	0.2518
		2	0.3349

		3	1.6944
		4	0.1352
**9**	**40**	1 (might be the ground state)	0.1252
		2	0.1987
		3	1.8394
		4	0.0572
**10**	**50**	1 (might be the ground state)	0.0631
		2	0.2572
		3	1.8004
		4	0.0331
**11**	**56**	1 (might be the ground state)	0.0919
		2	0.4129
		3	1.6224
		4	0.0424
**12**	**60**	1 (might be the ground state)	0.1445
		2	0.5417
		3	1.4634
		4	0.0641
**13**	**70**	1 (might be the ground state)	0.3326
		2	1.4496
		3	0.5882
		4	0.1972

Table S5. Tabulated potential energy difference between S_0 and S_1 states vs torsional angle ϕ' at S_1-MEP for monomer in THF solution.

**Serial No.**	**Dihedral Angle (ϕ') value (in degrees)**	**S_0-state Energy (in eV)**	**S_1-state Energy (in eV)**	**Difference in Energy (in eV)**
**1**	**55.5**	0<br>(S_0-MIN)	4.665<br>(FC)	4.665
**2**	**63.2**	0.97	3.527<br>(S_1-MIN)	2.557
**3**	**70**	1.967	3.545	1.578
**4**	**80**	2.079	3.636	1.557
**5**	**90**	2.270	3.799	1.529
**6**	**100**	1.999	2.779	0.780
**7**	**110**	2.047	2.846	0.799
**8**	**120**	2.089	2.899	0.810
**9**	**130**	2.203	3.014	0.811
**10**	**140**	2.313	3.171	0.858
**11**	**150**	2.378	3.373	0.995
**12**	**160**	1.867	3.564	1.697
**13**	**170**	1.770	3.770	2.000
**14**	**180**	1.649	3.986	2.337

Table S6. Tabulated oscillator strength *(f)* values along ϕ' torsional angle at S_1 -MEP of monomer in THF solution.

**Serial No.**	**Dihedral Angle (ϕ') value (in degrees)**	**Oscillator Strength (*f*) (in a.u.) ($S_1 \rightarrow S_0$)**
**1**	**63.2 (S_1-MIN)**	0.003833
**2**	**70**	0.003779
**3**	**80**	0.003626
**4**	**90**	0.003495
**5**	**100**	0.000201
**6**	**110**	0.001035
**7**	**120**	0.005226
**8**	**130**	0.012277
**9**	**140**	0.027464
**10**	**150**	0.054924
**11**	**160**	0.199575
**12**	**170**	0.260280
**13**	**180**	0.323050

Table S7. $\langle S^2 \rangle$ values for Excited state 1, Excited state 2 and Excited state 3 along ϕ' torsional angle at S_1-MEP of monomer in THF solution.

**Serial No.**	**Dihedral Angle (ϕ') (in degrees)**	**S_1 PES (States)**	**$\langle S^2 \rangle$ Values**
**1**	**55.4 (FC)**	1 (might be the ground state)	0.0516
		2	2.0326
		3	0.2550
		4	1.0529
**2**	**63.2 (S_1-MIN)**	1 (might be the ground state)	0.0391
		2	2.0283
		3	0.0395
		4	1.0537
**3**	**70**	1 (might be the ground state)	0.0401
		2	2.0293
		3	0.0407
		4	1.0509
**4**	**80**	1 (might be the ground state)	0.0414
		2	2.0300
		3	0.0427
		4	1.0437
**5**	**90**	1 (might be the ground state)	0.0429
		2	2.0310
		3	0.0448
		4	1.0304
**6**	**100**	1 (might be the ground state)	1.3150
		2	1.0948

		3	0.1517
		4	0.1518
**7**	**110**	1 (might be the ground state)	1.2329
		2	1.1632
		3	0.1513
		4	0.1569
**8**	**120**	1 (might be the ground state)	0.8739
		2	1.5063
		3	1.1542
		4	0.1591
**9**	**130**	1 (might be the ground state)	0.4750
		2	1.8865
		3	0.1555
		4	0.1622
**10**	**140**	1 (might be the ground state)	0.1713
		2	2.1666
		3	0.1573
		4	0.1668
**11**	**150**	1 (might be the ground state)	0.1281
		2	2.1838
		3	0.1599
		4	0.1772
**12**	**160**	1 (might be the ground state)	0.1095
		2	2.1641
		3	0.1612

		4	0.2841
**13**	**170**	1 (might be the ground state)	0.0999
		2	2.1477
		3	0.1653
		4	0.4852
**14**	**180**	1 (might be the ground state)	0.0955
		2	2.1371
		3	0.1777
		4	0.9247

Table S8. Tabulated potential energy difference between S_0 and S_1 states vs torsional angle ϴ' at S_1-MEP for monomer in THF solution.

Serial No.	Dihedral Angle (ϴ') value (in degrees)	S_0-state Energy (in eV)	S_1-state Energy (in eV)	Difference in Energy (in eV)
**1**	**6.3**	0 (S_0-MIN)	4.665 (FC)	4.665
**2**	**21.4**	0.97	3.527 (S_1-MIN)	2.557
**3**	**30**	2.002	3.545	1.543
**4**	**40**	2.146	3.615	1.469
**5**	**50**	2.768	3.421	0.653
**6**	**60**	2.480	3.161	0.681
**7**	**70**	2.237	2.972	0.735
**8**	**80**	2.056	2.855	0.829
**9**	**90**	1.941	2.805	0.864
**10**	**100**	1.819	2.811	0.992
**11**	**110**	1.511	2.849	1.338
**12**	**120**	1.437	2.905	1.468

**13**	**130**	1.413	3.002	1.589
**14**	**140**	1.443	3.151	1.708
**15**	**150**	1.535	3.356	1.821
**16**	**160**	1.695	3.619	1.924
**17**	**170**	0.827	4.789	3.962

Table S9. Tabulated oscillator strength *(f)* values along ϴ' torsional angle at S_1 -MEP of monomer in THF solution.

Serial No.	Dihedral Angle (ϴ') value (in degrees)	Oscillator Strength (*f*) (in a.u.) ($S_1 \rightarrow S_0$)
**1**	**21.4 (S_1-MIN)**	0.003833
**2**	**30**	0.00356
**3**	**40**	0.00321
**4**	**50**	0.00456
**5**	**60**	0.00094
**6**	**70**	0.00034
**7**	**80**	0.00283
**8**	**90**	0.01112
**9**	**100**	0.03682
**10**	**110**	0.13191
**11**	**120**	0.17358
**12**	**130**	0.20773
**13**	**140**	0.23717
**14**	**150**	0.26057
**15**	**160**	0.27697
**16**	**170**	0.86437

Table S10. **$\langle S^2 \rangle$ values for Excited state 1, Excited state 2 and Excited state 3 along ϴ' torsional angle at S_1-MEP of monomer in THF solution.**

**Serial No.**	**Dihedral Angle (ϴ') (in degrees)**	**S_1 PES (States)**	**$\langle S^2 \rangle$ Values**
**1**	**6.3 (FC)**	1 (might be the ground state)	0.0516
		2	2.0326
		3	0.2550
		4	1.0529
**2**	**21.4 (S_1-MIN)**	1 (might be the ground state)	0.0391
		2	2.0283
		3	0.0395
		4	1.0537
**3**	**30**	1 (might be the ground state)	0.0409
		2	2.0301
		3	0.0413
		4	1.0418
**4**	**40**	1 (might be the ground state)	0.0437
		2	2.0324
		3	0.0441
		4	0.9804
**5**	**50**	1 (might be the ground state)	0.8097
		2	1.5571
		3	0.1578
		4	0.1819
**6**	**60**	1 (might be the ground state)	1.1173

		2	1.2664
		3	0.1546
		4	0.1696
**7**	**70**	1 (might be the ground state)	1.4675
		2	0.9304
		3	0.1532
		4	0.1617
**8**	**80**	1 (might be the ground state)	1.0205
		2	1.3858
		3	0.1534
		4	0.1581
**9**	**90**	1 (might be the ground state)	0.5670
		2	1.8419
		3	0.1551
		4	0.1580
**10**	**100**	1 (might be the ground state)	0.2166
		2	2.1942
		3	0.1573
		4	0.1611
**11**	**110**	1 (might be the ground state)	0.1550
		2	2.2471
		3	0.1508
		4	0.1895
**12**	**120**	1 (might be the ground state)	0.1499
		2	2.2452

		3	0.1533
		4	0.2118
**13**	**130**	1 (might be the ground state)	0.1453
		2	2.2408
		3	0.1573
		4	0.2379
**14**	**140**	1 (might be the ground state)	0.1410
		2	2.2342
		3	0.1627
		4	0.2705
**15**	**150**	1 (might be the ground state)	0.1370
		2	2.2259
		3	0.1707
		4	0.3288
**16**	**160**	1 (might be the ground state)	0.1330
		2	2.2147
		3	0.1820
		4	0.9027
**17**	**170**	1 (might be the ground state)	0.0843
		2	2.0976
		3	0.3162
		4	1.0555

Figure S1. (a) Plot of potential energy profile along S_1-state i.e., S_1-MEP using Θ' torsion angle of THBDBA monomer in THF solution (calculated with SF-TDDFT/LRC-ωPBEh/cc-pVDZ/LR-PCM, THF) (b) Plot of oscillator strength value vs torsion angle (Θ') along S_1-MEP (c) Optimized geometry corresponding to S_1/S_0-MECI'' (Θ' = 105.3⁰) [Using penalty constrained optimization algorithm at S_1-MEP].

(a)

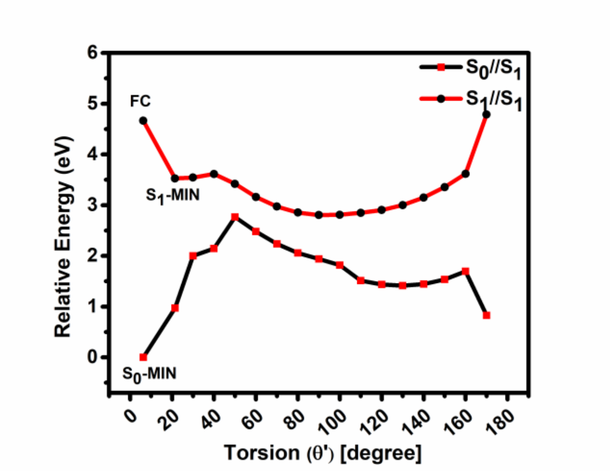

(b)

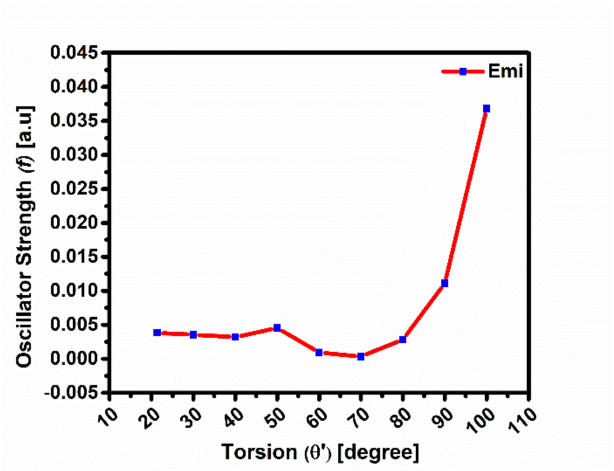

(c)

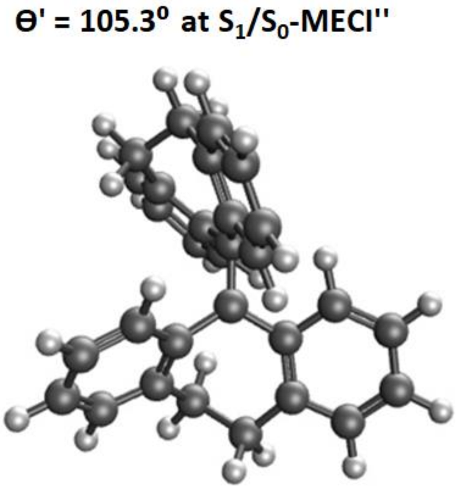

Figure S2. Molecular orbitals of THBDBA monomer at the S_0-MIN, S_1-MIN, S_1/S_0-MECI, S_1/S_0-MECI', and S_1/S_0-MECI'' which are calculated using SF-TDDFT with LRC-ωPBEh/cc-pVDZ. The response coefficients corresponding to each transition have been placed above the arrows.

Geometry	States	Transitions		
At S_0-Optimized	S_0	Ground (0.9850)		
	T_1	βHOMO 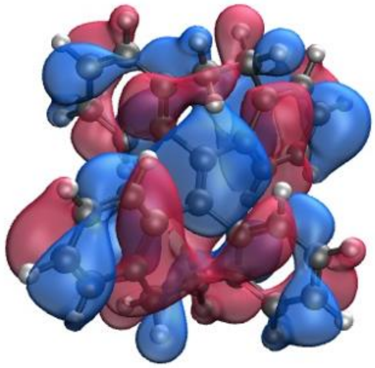	1.0000 →	αLUMO 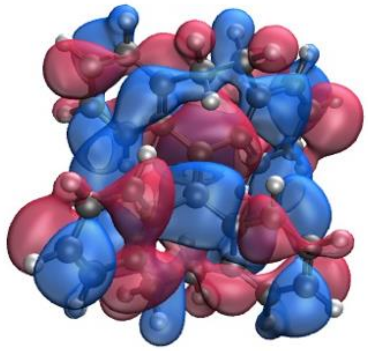
	S_1	βHOMO 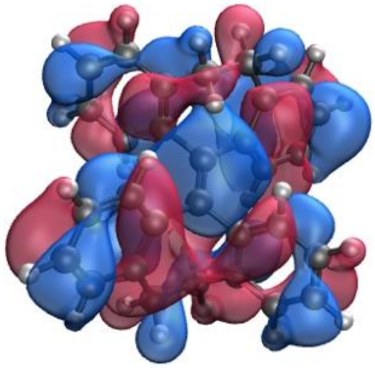	0.6580 →	βLUMO 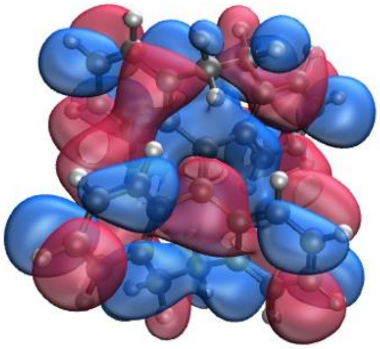
At S_1-Optimized	S_0	Ground (0.9875)		
	T_1	βHOMO 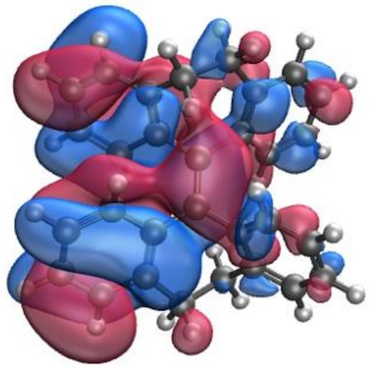	1.0000 →	αLUMO 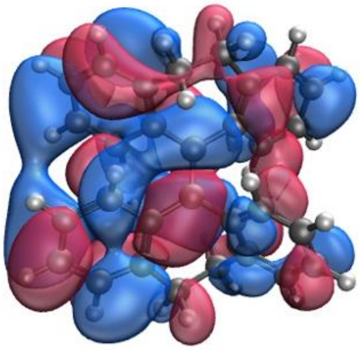

	S$_1$	**αHOMO** 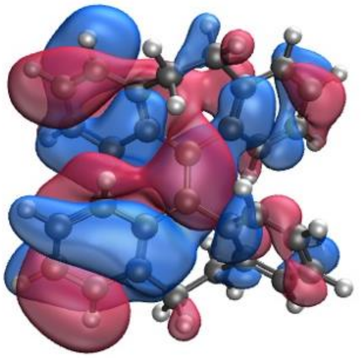	**0.6940** →	**αLUMO** 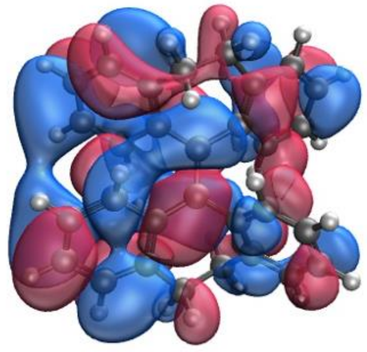
		**βHOMO** 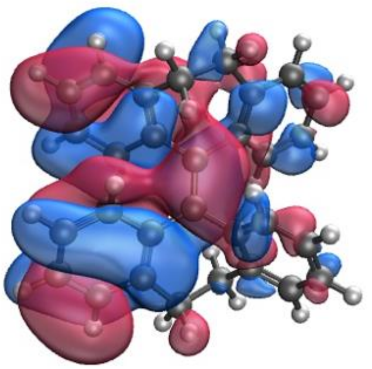	**0.6976** →	**βLUMO** 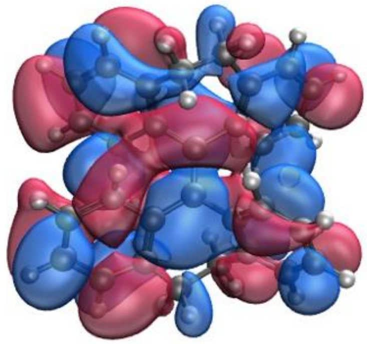
	S$_0$	**Ground (0.9695)**		
		**αHOMO** 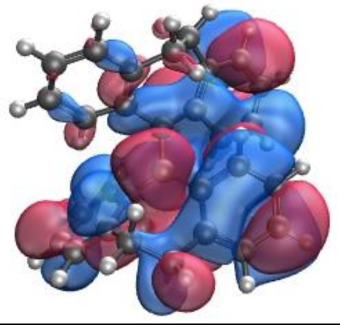	**0.1715** →	**αLUMO** 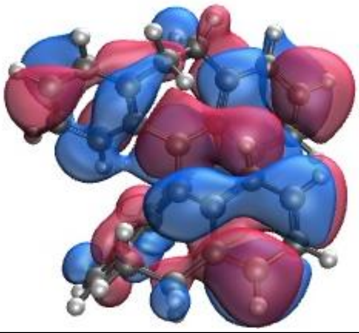
	T$_1$	**βHOMO**	**1.0000** →	**αLUMO**

S$_1$/S$_0$-MECI		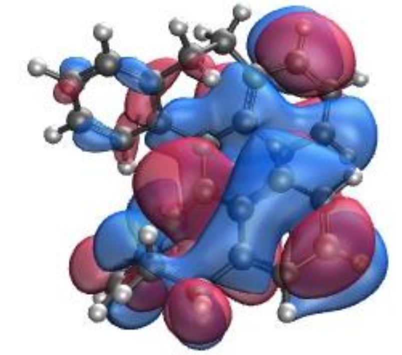		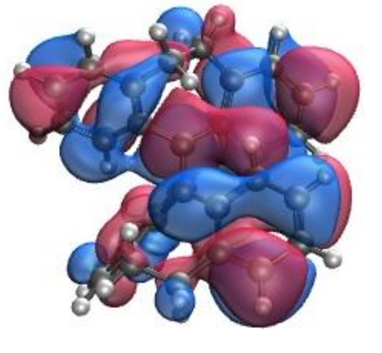
	S$_1$	αHOMO 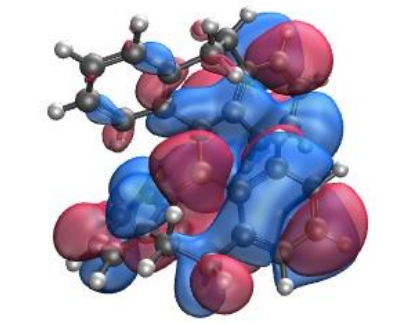	-0.6206 →	αLUMO 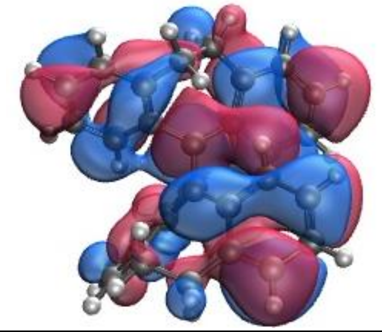
		βHOMO 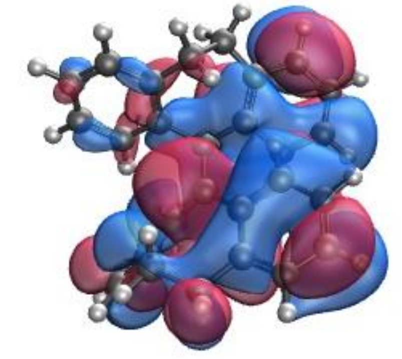	0.7507 →	βLUMO 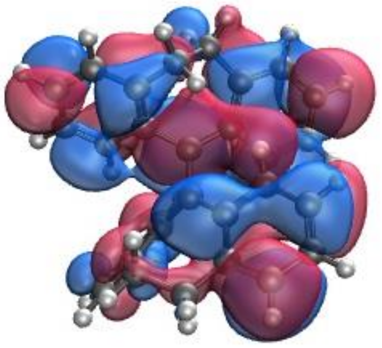
	T$_1$	αHOMO 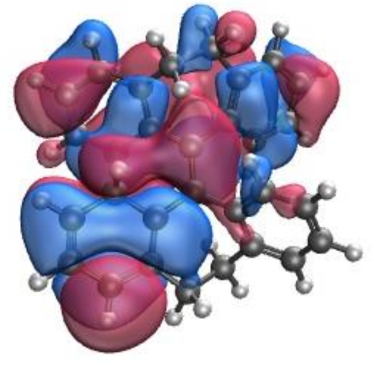	-0.4914 →	αLUMO 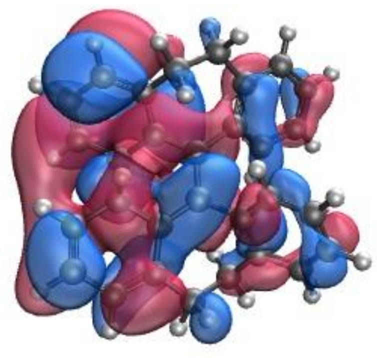
		βHOMO	0.8304 →	βLUMO

S_1/S_0-MECI'		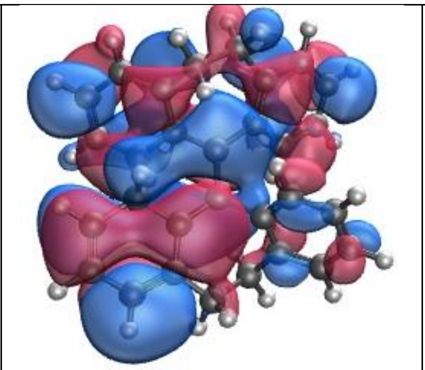		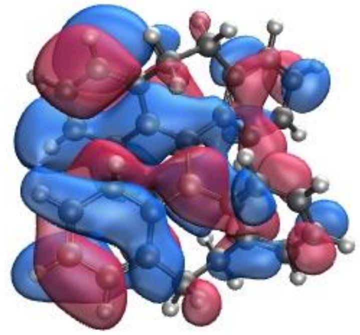
		αHOMO 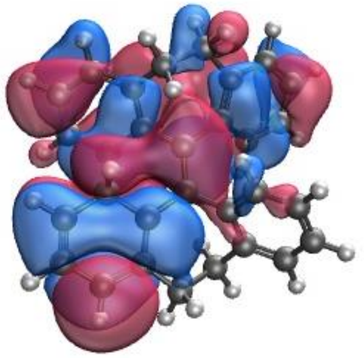	-0.6357 →	αLUMO 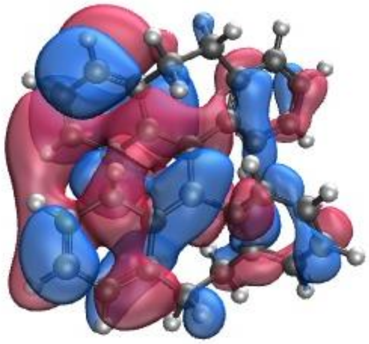
	S_1	βHOMO 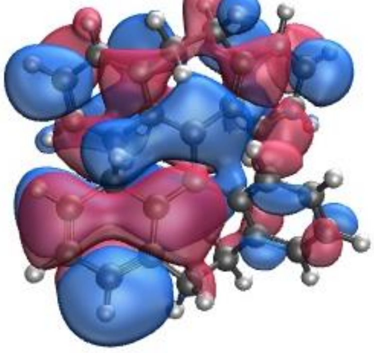	0.6503 →	αLUMO 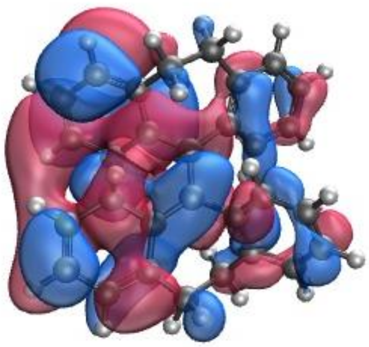
		βHOMO 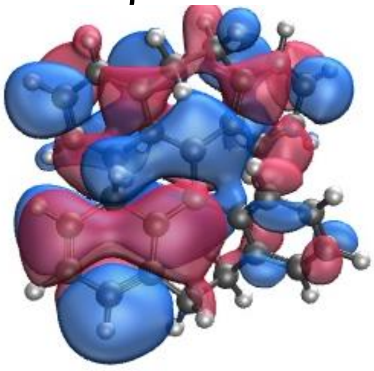	-0.3586 →	βLUMO 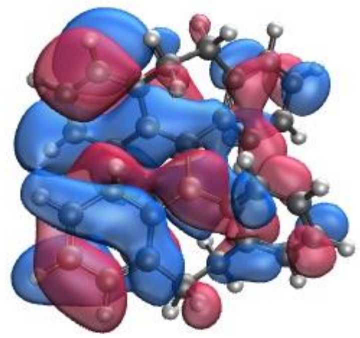
	T_1	αHOMO		αLUMO

**S_1/S_0-MECI''**		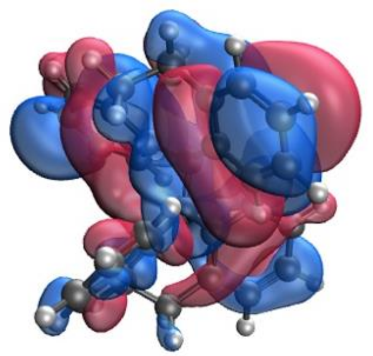	**0.2466** →	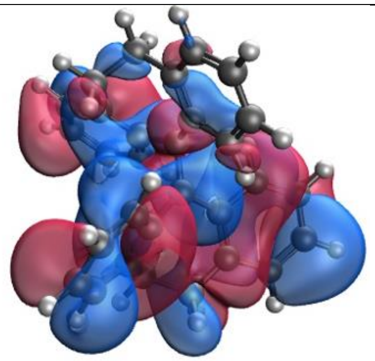
		**βHOMO** 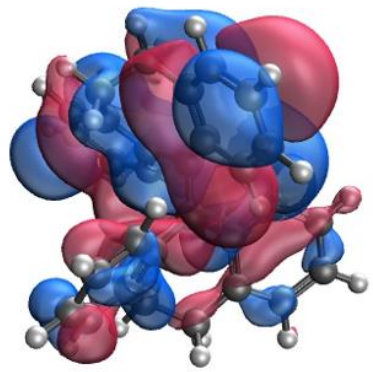	**0.9206** →	**βLUMO** 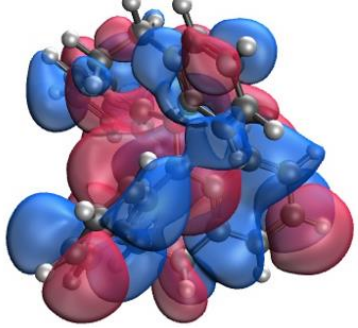
	**S_1**	**αHOMO** 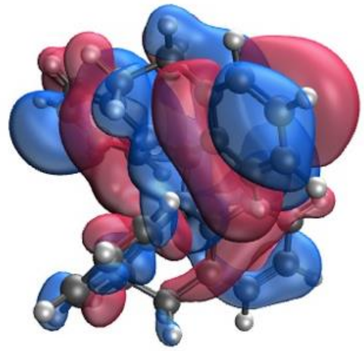	**0.2251** →	**αLUMO** 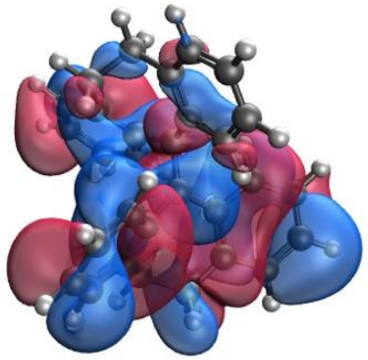
		**βHOMO** 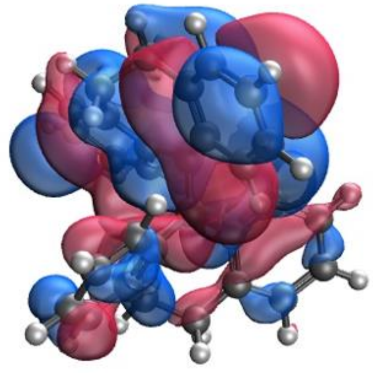	**0.9356** →	**αLUMO** 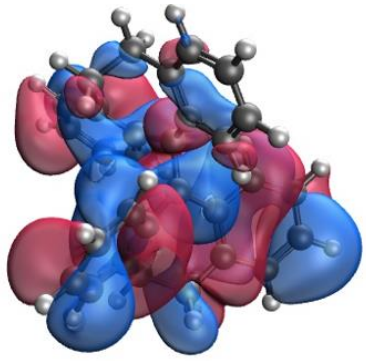

Table S11. Tabulated potential energy difference between S_0 and S_1 states vs torsional angle ϕ at S_1-MEP for dimer in THF solution.

**Serial No.**	**Dihedral Angle (ϕ) value (in degrees)**	**S_0-state Energy (in eV)**	**S_1-state Energy (in eV)**	**Difference in Energy (in eV)**
**1**	**-54.5**	0 (S_0-MIN)	4.638 (FC)	4.638
**2**	**-52.3**	0.658	3.956 (S_1-MIN)	3.298
**3**	**-50**	1.051	3.892	2.841
**4**	**-40**	1.788	3.634	1.846
**5**	**-30**	1.959	3.520	1.561
**6**	**-20**	2.227	3.652	1.425
**7**	**-60**	1.929	3.527	1.598

Table S12. Tabulated oscillator strength *(f)* values along ϕ torsional angle at S_1 -MEP of dimer in THF solution.

**Serial No.**	**Dihedral Angle (ϕ) value (in degrees)**	**Oscillator Strength (*f*) (in a.u.) ($S_1 \rightarrow S_0$)**
**1**	**-52.3 (S_1-MIN)**	0.5641
**2**	**-50**	0.2325
**3**	**-40**	0.0096
**4**	**-30**	0.0038
**5**	**-20**	0.0056
**6**	**-60**	0.0041

Table S13. ⟨S^2⟩ values for Excited state 1, Excited state 2 and Excited state 3 along ϕ torsional angle at S_1-MEP of dimer in THF solution.

Serial No.	Dihedral Angle (ϕ) (in degrees)	S_1 PES (States)	⟨S^2⟩ Values
**1**	**-54.57 (FC)**	1 (might be the ground state)	0.0526
		2	2.0359
		3	0.2506
		4	1.0538
**2**	**-52.35 (S_1-MIN)**	1 (might be the ground state)	0.0842
		2	2.1211
		3	0.1957
		4	1.0831
**3**	**-50**	1 (might be the ground state)	0.0911
		2	2.0913
		3	0.2436
		4	1.0251
**4**	**-40**	1 (might be the ground state)	0.0339
		2	1.9938
		3	0.0765
		4	1.0644
**5**	**-30**	1 (might be the ground state)	0.0403
		2	2.0292
		3	0.0405
		4	1.0516
**6**	**-20**	1 (might be the ground state)	0.0512
		2	2.0282

		3	0.0590
		4	0.5388
**7**	**-60**	1 (might be the ground state)	0.0384
		2	2.0277
		3	0.0391
		4	1.0579

S1. Cartesian coordinates of S_0-MIN, S_1-MIN, S_1/S_0-MECI, S_1/S_0-MECI', and S_1/S_0-MECI'' optimized structures for THBDBA monomer in THF

S_0-MIN of THBDBA monomer in THF

1	C	2.0207994336	2.7535100624	8.1085026973
2	H	1.6075189628	3.7408611716	8.3630940974
3	H	3.1063778825	2.7996063032	8.3029116127
4	C	1.7786257043	2.4975570418	6.6255644140
5	H	0.6931226424	2.5582760148	6.4321839748
6	C	2.2720295529	1.1949630481	6.0285982855
7	C	2.0388416358	1.0389409699	4.6549653774
8	H	1.5294820238	1.8484085396	4.1227683428
9	C	2.4299739313	-0.0941026866	3.9542922465
10	H	2.2253874566	-0.1759071667	2.8839040584
11	C	3.0761816657	-1.1252042514	4.6370487097
12	H	3.3750433793	-2.0381872908	4.1163328049
13	C	3.3281928934	-0.9879118063	5.9922390917
14	H	3.8113031148	-1.8045581436	6.5304194440
15	C	2.9510164461	0.1630343409	6.7183811772
16	C	3.2118913249	0.1654469899	8.1847550208
17	C	2.0128869955	0.4317383824	9.0271529303
18	C	1.4675361081	-0.5682971427	9.8406822337
19	H	1.9481737951	-1.5493561202	9.8736948242
20	C	0.3219716429	-0.3232840521	10.5931574123
21	H	-0.0967616125	-1.1144545149	11.2202387704
22	C	-0.2861588999	0.9302867337	10.5435374917
23	H	-1.1832946059	1.1309225162	11.1346982805
24	C	0.2573709883	1.9317684658	9.7399938843
25	H	-0.2090692108	2.9211152648	9.7110971235

26	C	1.4025333121	1.6965409859	8.9776988923
27	C	5.5886652590	-2.7534843781	8.7887776644
28	H	6.0018634851	-3.7408328946	8.5340461554
29	H	4.5030341415	-2.7995639366	8.5946544542
30	C	5.8312219694	-2.4975865425	10.2716606666
31	H	6.9167782653	-2.5582933095	10.4647520265
32	C	5.3379781708	-1.1950229271	10.8688165070
33	C	5.5715274622	-1.0390859518	12.2423951308
34	H	6.0809971511	-1.8485969066	12.7744223610
35	C	5.1806215915	0.0939354848	12.9432262578
36	H	5.3854937150	0.1756786102	14.0135645290
37	C	4.5342816813	1.1250955588	12.2606941106
38	H	4.2355941780	2.0380619996	12.7815421972
39	C	4.2819049625	0.9878897981	10.9055654309
40	H	3.7986823179	1.8045781581	10.3675615001
41	C	4.6588428774	-0.1630381068	10.1792491061
42	C	4.3975926055	-0.1654098494	8.7129238709
43	C	5.5963623748	-0.4316794970	7.8702137645
44	C	6.1414890391	0.5683893747	7.0565910069
45	H	5.6608376750	1.5494490567	7.0237486428
46	C	7.2868507555	0.3234138163	6.3037895525
47	H	7.7054116056	1.1146109471	5.6766284285
48	C	7.8949899768	-0.9301637028	6.3531928266
49	H	8.7919671064	-1.1307787694	5.7617839105
50	C	7.3516659918	-1.9316767186	7.1568424481
51	H	7.8181119806	-2.9210245952	7.1855744825
52	C	6.2067036628	-1.6964809726	7.9194549477
53	H	2.2269729879	3.3231958375	6.0474133141
54	H	5.3830429069	-3.3232565491	10.8498973888

S_1-MIN of THBDBA monomer in THF

1	C	1.9073836048	2.9016012065	7.8522278358
2	H	1.4176870580	3.8696944172	8.0353688548
3	H	2.9879171247	3.0509494063	8.0267003629
4	C	1.6819646228	2.5190183059	6.4023638868
5	H	0.5951099019	2.4680750333	6.2142752015
6	C	2.2861836958	1.2220487317	5.9180413130
7	C	2.0952465648	0.9611293719	4.5513333811
8	H	1.5822020741	1.7210793885	3.9533321793

9	C	2.5150086859	-0.2115561282	3.9426955532
10	H	2.3445243199	-0.3739150584	2.8756163782
11	C	3.1326138294	-1.1835979503	4.7283941171
12	H	3.4419599599	-2.1393023192	4.2978063215
13	C	3.3451476172	-0.9360162539	6.0748794328
14	H	3.8046808080	-1.7179978168	6.6799453507
15	C	2.9706234985	0.2727513953	6.7076280718
16	C	3.2206798666	0.3211801262	8.1856487698
17	C	2.1359587811	0.6837822863	9.0388238125
18	C	1.8995625185	-0.0760816553	10.2582661542
19	H	2.2039713073	-1.1259950525	10.2550140339
20	C	0.8138313227	0.2805186930	11.1061063264
21	H	0.5811454873	-0.3583395238	11.9618186447
22	C	0.0907667037	1.4206690459	10.8673792410
23	H	-0.7276227857	1.7102546069	11.5310385909
24	C	0.4275683394	2.2504128582	9.7628115174
25	H	-0.0732112666	3.2167015587	9.6520726580
26	C	1.4123316728	1.9045247098	8.8618491412
27	C	5.8595390300	-2.8085213903	8.6902761535
28	H	6.3808552530	-3.7357317554	8.4106035273
29	H	4.7757807700	-2.9988782445	8.6031813357
30	C	6.2109654880	-2.4555053643	10.1327375191
31	H	7.2610285725	-2.1123623080	10.1554980457
32	C	5.3462404026	-1.4202105010	10.8222632459
33	C	5.2757232497	-1.5335755521	12.2017710999
34	H	5.8277247860	-2.3510412186	12.6750898285
35	C	4.5227187208	-0.6643822875	13.0239932254
36	H	4.5351422124	-0.7954844303	14.1085668919
37	C	3.8104946741	0.3508894672	12.4406340142
38	H	3.2538476108	1.0717954726	13.0447793683
39	C	3.7739417809	0.4736360252	11.0279410891
40	H	3.5917107722	1.4811082308	10.6437300179
41	C	4.5634317357	-0.4092770549	10.1647490109
42	C	4.4092009616	-0.2604194403	8.7434738541
43	C	5.5906159909	-0.4703499752	7.8567546460
44	C	6.0419918369	0.6032434377	7.0731878905
45	H	5.5213077620	1.5621855626	7.1360575880
46	C	7.1344266228	0.4670389243	6.2226573215
47	H	7.4666287521	1.3196427060	5.6247608509
48	C	7.7977887025	-0.7560367629	6.1363526023

49	H	8.6520255479	-0.8769906068	5.4653812882
50	C	7.3673942066	-1.8230902713	6.9210962820
51	H	7.8869449339	-2.7845394248	6.8679076054
52	C	6.2753112587	-1.6925801501	7.7824875438
53	H	2.0537657407	3.3351842509	5.7604947349
54	H	6.1806797716	-3.3673000318	10.7490321698

S_1/S_0-MECI of THBDBA monomer in THF

1	C	2.2694193081	3.0215370290	7.3746116287
2	H	2.0522287431	4.0967305607	7.3010210854
3	H	3.3035377878	2.8718479764	7.0198094250
4	C	1.3049211197	2.2313422748	6.4898378726
5	H	0.3580309573	2.0828968641	7.0382467804
6	C	1.8806047459	0.9127936734	6.0506741758
7	C	1.6774750106	0.5446699160	4.7319411206
8	H	1.0619689318	1.1934348837	4.1028155154
9	C	2.2472567292	-0.6215709735	4.1623759639
10	H	2.0097507287	-0.8905440244	3.1299056595
11	C	3.0835031627	-1.3983048962	4.9140897413
12	H	3.5346459743	-2.3039345708	4.5012832597
13	C	3.3986883851	-1.0269952549	6.2598495728
14	H	3.6516116142	-1.8583032702	6.9248828921
15	C	2.7001367309	0.0975948473	6.8991479474
16	C	3.0541315235	0.2988592429	8.2678230094
17	C	2.3677613150	1.2556780945	9.2017100424
18	C	2.0520269409	0.9254525709	10.5333563940
19	H	2.1917874025	-0.0914069790	10.8871877543
20	C	1.5370454920	1.8539040562	11.4329106371
21	H	1.3027651472	1.5352364965	12.4517754168
22	C	1.3227240079	3.1681356872	11.0348931810
23	H	0.9294122159	3.9115109070	11.7325800495
24	C	1.6033834387	3.5141376368	9.7178667746
25	H	1.4227025899	4.5366705372	9.3734571772
26	C	2.1085942717	2.5884277595	8.8003472435
27	C	7.1944861896	-1.0273426820	9.7255600068
28	H	8.1963885555	-0.5690015947	9.6697149461
29	H	7.3574690826	-2.0626992460	10.0724502114
30	C	6.3660792354	-0.2873294573	10.7707188319
31	H	6.1184815641	0.7239568885	10.4080789413

32	C	5.1250395725	-1.0806426603	11.0326966647
33	C	4.9722984691	-1.8425011087	12.1937770685
34	H	5.7274830846	-1.7641775901	12.9817417664
35	C	3.8883511351	-2.7022882062	12.3536141265
36	H	3.7850572408	-3.2893389386	13.2696984323
37	C	2.9510849933	-2.8238060270	11.3278178637
38	H	2.1100735034	-3.5148178252	11.4256673984
39	C	3.0894525126	-2.0584770268	10.1751187322
40	H	2.3512290808	-2.1437016041	9.3723690390
41	C	4.1572639738	-1.1554747879	10.0206305359
42	C	4.2288667737	-0.3959093662	8.7482668011
43	C	5.3729997645	-0.5282308873	7.9006200302
44	C	5.2065592567	-0.2321588607	6.4645494695
45	H	4.7839426775	0.7440957443	6.2022971274
46	C	6.1648684031	-0.7311300420	5.5283721004
47	H	6.0130491736	-0.5156372429	4.4673382857
48	C	7.2551019533	-1.4440828086	5.9510522107
49	H	7.9732412711	-1.8521073897	5.2358572898
50	C	7.5174471249	-1.5275835770	7.3390804276
51	H	8.4711582363	-1.9522447821	7.6682002093
52	C	6.6483364095	-1.0479073851	8.3073928351
53	H	1.0523722566	2.8080740948	5.5875394691
54	H	6.9561666922	-0.1772999864	11.6922747390

S_1/S_0-MECI' of THBDBA monomer in THF

1	C	1.8199030180	2.8255097233	7.8326682651
2	H	1.3086406673	3.7810794633	8.0220131068
3	H	2.8966002419	2.9893464183	8.0226801251
4	C	1.6246617261	2.4516976984	6.3760659033
5	H	0.5421628716	2.3911709104	6.1680915477
6	C	2.2572725231	1.1634364371	5.9039344603
7	C	2.0799822801	0.8907222232	4.5368606316
8	H	1.5523659592	1.6356732578	3.9324198878
9	C	2.5343384923	-0.2718033529	3.9346381293
10	H	2.3776197668	-0.4421045960	2.8667014346
11	C	3.1636437935	-1.2271502708	4.7321841980
12	H	3.4960982435	-2.1790107862	4.3099186936
13	C	3.3458189486	-0.9735919763	6.0819906380
14	H	3.8017417443	-1.7503573388	6.6975433800

15	C	2.9544720835	0.2346146809	6.7073705737
16	C	3.2215994937	0.2963135682	8.1809295866
17	C	2.1610066191	0.6311690009	9.0507390577
18	C	1.9341016923	-0.1223731103	10.2812973547
19	H	2.1597404878	-1.1909904290	10.2303520883
20	C	0.8399293819	0.2217634655	11.1339003585
21	H	0.6596006689	-0.4052408056	12.0119184364
22	C	0.0639836993	1.3188128430	10.8903824225
23	H	-0.7419243059	1.6188586548	11.5612035815
24	C	0.3663902456	2.1092431578	9.7421131833
25	H	-0.1865336397	3.0450316146	9.6048176544
26	C	1.3527576170	1.8152716780	8.8370241857
27	C	6.0226277294	-2.7367007771	8.8007806965
28	H	6.6021754293	-3.6402321781	8.5611902480
29	H	4.9551306931	-3.0151535263	8.7900250928
30	C	6.4181008642	-2.2276673640	10.1861820539
31	H	7.3980423738	-1.7254916983	10.0950272157
32	C	5.4473657694	-1.2833138983	10.8677029088
33	C	5.3953275975	-1.3677067603	12.2266158919
34	H	6.0329613897	-2.0869349538	12.7451852117
35	C	4.5065648376	-0.5425095720	12.9935089037
36	H	4.5720593863	-0.5681706067	14.0863780480
37	C	3.6030260462	0.2729324411	12.4092850214
38	H	2.9443060236	0.9083443562	13.0050202642
39	C	3.5415549700	0.3819309625	10.9472465247
40	H	3.5188446101	1.4415534613	10.6309452605
41	C	4.5480803978	-0.3910335535	10.1506279615
42	C	4.4193321534	-0.2697547305	8.7640582014
43	C	5.5944598149	-0.4640906570	7.8573131540
44	C	5.9871491438	0.6079939011	7.0435954689
45	H	5.4358738974	1.5493862607	7.0999428306
46	C	7.0583602501	0.4872912100	6.1642874730
47	H	7.3480757613	1.3377225752	5.5420576902
48	C	7.7463412898	-0.7211412315	6.0735201470
49	H	8.5779013018	-0.8338295351	5.3732408710
50	C	7.3773398892	-1.7816136427	6.8970593588
51	H	7.9291058624	-2.7251821476	6.8523036727
52	C	6.3180971098	-1.6620560199	7.8004430343
53	H	1.9997729435	3.2772385688	5.7481407247
54	H	6.5765106052	-3.0788663226	10.8654530668

S_1/S_0-MECI'' of THBDBA monomer in THF

1	C	0.6618887348	1.8018777915	7.2878174810
2	H	-0.3185252166	2.3022495752	7.3271515270
3	H	1.0392673711	1.9369836468	6.2581246664
4	C	0.4881831864	0.2880967587	7.5404773610
5	H	0.5587340816	0.1098173829	8.6280675661
6	C	1.5075901488	-0.5394567533	6.8193623506
7	C	1.1001240743	-1.5285834072	5.9378829480
8	H	0.0238043834	-1.6866869809	5.8078325735
9	C	2.0022540536	-2.3544601460	5.2478725036
10	H	1.6391036395	-3.1529845992	4.5971237920
11	C	3.3556396218	-2.1054205151	5.3992087743
12	H	4.0947742323	-2.6979378880	4.8517265501
13	C	3.7939065731	-1.0524530348	6.2083235023
14	H	4.8583667960	-0.8132525593	6.1905034522
15	C	2.9047747490	-0.2389249122	6.9716098409
16	C	3.4465305864	0.7748602483	7.8729976124
17	C	2.8543932903	1.8958004308	8.5677424942
18	C	3.6076599832	2.5764268700	9.5646927321
19	H	4.6058902892	2.2156373290	9.8306861853
20	C	3.1387361051	3.7078808071	10.2333797281
21	H	3.7777923680	4.1739240227	10.9904444761
22	C	1.8910688647	4.2394132222	9.9422876678
23	H	1.5121944426	5.1279049820	10.4515748494
24	C	1.1325080723	3.5873560034	8.9664484681
25	H	0.1360438037	3.9702414855	8.7172661650
26	C	1.5725501261	2.4547404037	8.2873858415
27	C	6.9453974633	-2.0482124885	9.0650921254
28	H	7.1608346334	-2.0529766985	10.1486628701
29	H	7.7753689907	-2.5928333604	8.5909418556
30	C	5.6417736674	-2.7730895186	8.7879165588
31	H	5.7808963063	-3.8486928799	8.9597304906
32	C	4.5639702648	-2.2704970590	9.7002071222
33	C	4.0222757250	-3.1304478852	10.6601959429
34	H	4.3691019591	-4.1656727760	10.6954062168
35	C	3.0722817484	-2.6858373887	11.5717816281
36	H	2.6870433213	-3.3732698898	12.3289941158
37	C	2.6068949247	-1.3736518289	11.5170460479
38	H	1.8594429442	-1.0175450339	12.2281127511

39	C	3.0708169163	-0.5290668816	10.5193031779
40	H	2.6838544238	0.4847192009	10.4332823927
41	C	4.0692233096	-0.9546921182	9.6279777981
42	C	4.4487229535	-0.0179623810	8.5206259459
43	C	5.9013741138	0.1620751226	8.1843844417
44	C	6.1065625644	1.4420581248	7.6458664171
45	H	5.1957328055	1.9614308096	7.3322936014
46	C	7.3810684902	1.9892739259	7.5370589735
47	H	7.5227937938	2.9899384481	7.1263669523
48	C	8.4604873250	1.2260603720	7.9511865870
49	H	9.4756161276	1.6237434376	7.8707528837
50	C	8.2763221298	-0.0641035646	8.4520954505
51	H	9.1489971290	-0.6584576322	8.7325844734
52	C	7.0096588257	-0.6274634041	8.5815867589
53	H	-0.5165258121	-0.0373317103	7.2279285455
54	H	5.3552210549	-2.6505084156	7.7315226468

S2. Cartesian coordinates of S_0-MIN and S_1-MIN optimized structures for THBDBA dimer in THF

S_0-MIN of THBDBA dimer in THF

1	C	1.7346542742	2.5117242529	7.9625102929
2	H	1.2256360655	3.4649031227	8.1693758113
3	H	2.8111773179	2.6745459196	8.1418866554
4	C	1.5147485800	2.1544662991	6.4976495015
5	H	1.8762766973	2.9894553143	5.8739757627
6	H	0.4288221763	2.0938444711	6.3074897425
7	C	2.1396680145	0.8791294901	5.9698005950
8	C	1.9140858061	0.6187436688	4.6108282693
9	H	1.3109310206	1.3329963203	4.0417462694
10	C	2.4268129474	-0.5005521494	3.9692568468
11	H	2.2240086443	-0.6662511755	2.9084649857
12	C	3.1930221350	-1.4100271756	4.6989134642
13	H	3.5913489189	-2.3104944262	4.2252781222
14	C	3.4353429179	-1.1698628812	6.0415003026
15	H	4.0097697636	-1.8958563719	6.6184540096
16	C	2.9328855325	-0.0308964012	6.7080422333
17	C	3.1910251977	0.0732964964	8.1714892269
18	C	1.9685229235	0.2539064976	9.0034686093
19	C	1.5304652595	-0.7511718198	9.8736358374

20	H	2.1094311465	-1.6748486100	9.9553992205
21	C	0.3699613460	-0.5815557355	10.6244883815
22	H	0.0373872257	-1.3754339758	11.2980979117
23	C	-0.3617745944	0.6000425641	10.5148609705
24	H	-1.2697404785	0.7418660716	11.1060603151
25	C	0.0725431065	1.6052299450	9.6520377624
26	H	-0.4909240920	2.5398016038	9.5745752999
27	C	1.2313268340	1.4449220441	8.8910170629
28	C	5.8967430177	-2.5216393948	8.9205357486
29	H	6.4214305114	-3.4655428888	8.7105297384
30	H	4.8238099269	-2.6994767400	8.7332567353
31	C	6.1033164944	-2.1692311562	10.3881536090
32	H	5.7455706498	-3.0098321565	11.0065517574
33	H	7.1872014432	-2.0975173096	10.5860775297
34	C	5.4607788972	-0.9028695731	10.9165350519
35	C	5.6764385299	-0.6471401657	12.2781711400
36	H	6.2884771352	-1.3553474782	12.8452049016
37	C	5.1416675315	0.4580113467	12.9260405094
38	H	5.3353404033	0.6191643266	13.9892051687
39	C	4.3633399457	1.3585654077	12.1985218725
40	H	3.9451165768	2.2483736154	12.6756649636
41	C	4.1348781583	1.1248879959	10.8521318419
42	H	3.5518437479	1.8463178199	10.2784715314
43	C	4.6590439650	0.0000930868	10.1779803829
44	C	4.4048937458	-0.0990727560	8.7123342865
45	C	5.6294768614	-0.2674400200	7.8802285741
46	C	6.0549109861	0.7421236079	7.0078664670
47	H	5.4538058530	1.6495194043	6.9093706571
48	C	7.2322242645	0.6000320698	6.2775396465
49	H	7.5545093782	1.3998048462	5.6057068267
50	C	7.9938270169	-0.5606026561	6.4069964502
51	H	8.9177562459	-0.6796172649	5.8358456680
52	C	7.5668310006	-1.5749766615	7.2623477662
53	H	8.1514270747	-2.4953150688	7.3519032255
54	C	6.3907612698	-1.4420493066	8.0021520193
55	C	2.0590252719	9.8386898835	8.0412050457
56	H	1.6840252719	10.7120898835	8.2795050457
57	H	3.0160252719	9.8549898835	8.2524050457
58	C	1.8921252719	9.6215898835	6.6320050457
59	H	2.3698252719	10.3351898835	6.1606050457

60	H	0.9399252719	9.7255898835	6.4242050457
61	C	2.3613252719	8.2633898835	6.0387050457
62	C	2.1149252719	8.1307898835	4.6716050457
63	H	1.7539252719	8.8598898835	4.2005050457
64	C	2.3824252719	6.9715898835	3.9950050457
65	H	2.1919252719	6.9079898835	3.0768050457
66	C	2.9315252719	5.8986898835	4.6613050457
67	H	3.1124252719	5.0966898835	4.2055050457
68	C	3.2120252719	6.0155898835	6.0044050457
69	H	3.5993252719	5.2862898835	6.4546050457
70	C	2.9401252719	7.1800898835	6.7165050457
71	C	3.2063252719	7.2163898835	8.1830050457
72	C	1.9972252719	7.4705898835	9.0160050457
73	C	1.4937252719	6.4814898835	9.8499050457
74	H	1.9361252719	5.6546898835	9.9084050457
75	C	0.3547252719	6.6957898835	10.5936050457
76	H	0.0207252719	6.0160898835	11.1503050457
77	C	-0.2907747281	7.9029898835	10.5202050457
78	H	-1.0693747281	8.0500898835	11.0253050457
79	C	0.2000252719	8.8980898835	9.7087050457
80	H	-0.2409747281	9.7269898835	9.6786050457
81	C	1.3433252719	8.7000898835	8.9280050457
82	C	5.5587252719	4.3180898835	8.8591050457
83	H	5.9337252719	3.4446898835	8.6208050457
84	H	4.6016252719	4.3017898835	8.6478050457
85	C	5.7256252719	4.5351898835	10.2683050457
86	H	5.2478252719	3.8215898835	10.7397050457
87	H	6.6777252719	4.4311898835	10.4761050457
88	C	5.2563252719	5.8933898835	10.8616050457
89	C	5.5027252719	6.0259898835	12.2287050457
90	H	5.8637252719	5.2968898835	12.6998050457
91	C	5.2352252719	7.1851898835	12.9053050457
92	H	5.4257252719	7.2487898835	13.8235050457
93	C	4.6862252719	8.2580898835	12.2390050457
94	H	4.5053252719	9.0600898835	12.6947050457
95	C	4.4056252719	8.1411898835	10.8959050457
96	H	4.0183252719	8.8704898835	10.4457050457
97	C	4.6776252719	6.9766898835	10.1838050457
98	C	4.4113252719	6.9403898835	8.7173050457
99	C	5.6204252719	6.6861898835	7.8843050457

100	C	6.1240252719	7.6752898835	7.0504050457
101	H	5.6815252719	8.5020898835	6.9919050457
102	C	7.2629252719	7.4609898835	6.3067050457
103	H	7.5969252719	8.1406898835	5.7500050457
104	C	7.9085252719	6.2537898835	6.3801050457
105	H	8.6870252719	6.1066898835	5.8750050457
106	C	7.4176252719	5.2586898835	7.1916050457
107	H	7.8587252719	4.4297898835	7.2217050457
108	C	6.2744252719	5.4566898835	7.9723050457

S_1-MIN of THBDBA dimer in THF

1	C	1.5953799274	2.6184108814	7.8733605640
2	H	1.0386864554	3.5466985135	8.0692081729
3	H	2.6631962196	2.8311379218	8.0539839870
4	C	1.3934155609	2.2248535700	6.4206370511
5	H	1.7251406325	3.0580024691	5.7768629222
6	H	0.3117152505	2.1185940911	6.2285546539
7	C	2.0773244743	0.9628103372	5.9381421553
8	C	1.8544036154	0.6524882790	4.5996356647
9	H	1.1889775460	1.3063814737	4.0266421194
10	C	2.4494502979	-0.4358921140	3.9557355268
11	H	2.2368262484	-0.6368603024	2.9030181336
12	C	3.3139829636	-1.2455368554	4.6812846461
13	H	3.7867700991	-2.1153133647	4.2184564726
14	C	3.5756514969	-0.9570510469	6.0127023585
15	H	4.1985440360	-1.6497937790	6.5732670616
16	C	2.9611058036	0.1349939115	6.7093315939
17	C	3.1734718895	0.2091572128	8.1484117887
18	C	1.9867517231	0.4087240017	8.9778741296
19	C	1.6361842054	-0.5413950481	9.9686389114
20	H	2.2780322887	-1.4104768084	10.1252723401
21	C	0.4526615875	-0.4206686953	10.6904687371
22	H	0.1883846933	-1.1872541458	11.4236382292
23	C	-0.3826812875	0.6750123491	10.4881280280
24	H	-1.3051214893	0.7826542749	11.0640721871
25	C	-0.0145537243	1.6559256771	9.5615867167
26	H	-0.6372748175	2.5471047390	9.4384993040
27	C	1.1493657113	1.5399530733	8.8108023575
28	C	6.0199068397	-2.6283765839	9.0001522645

29	H	6.5874550701	-3.5480033753	8.7950594278
30	H	4.9556967756	-2.8485117605	8.8069239500
31	C	6.2058063215	-2.2548616104	10.4604423057
32	H	5.8644879852	-3.0955770505	11.0894048802
33	H	7.2853957556	-2.1530614924	10.6664128503
34	C	5.5192802817	-0.9972694335	10.9514651571
35	C	5.7430403967	-0.6959148470	12.2926840701
36	H	6.4097938658	-1.3532024151	12.8602388646
37	C	5.1468614265	0.3860909396	12.9448635897
38	H	5.3578494192	0.5790926358	13.9993479858
39	C	4.2826775612	1.2005965812	12.2239825777
40	H	3.8071605957	2.0660105089	12.6924752263
41	C	4.0235118275	0.9222319827	10.8895994935
42	H	3.3976037727	1.6175200400	10.3356524093
43	C	4.6352511981	-0.1660222245	10.1866328187
44	C	4.4209209061	-0.2299191448	8.7447625782
45	C	5.6077329093	-0.4124903677	7.9155498571
46	C	5.9472573608	0.5442933699	6.9252026280
47	H	5.2911363558	1.4017821379	6.7650564892
48	C	7.1405186455	0.4481500786	6.2148694769
49	H	7.3968084182	1.2218600897	5.4862951779
50	C	7.9941935523	-0.6319955339	6.4219195649
51	H	8.9238650048	-0.7209091048	5.8544978342
52	C	7.6331877727	-1.6253043234	7.3392352788
53	H	8.2679812050	-2.5082691049	7.4594129814
54	C	6.4624134552	-1.5328283801	8.0809431547
55	C	2.0593817391	9.8389375513	8.0412852092
56	H	1.6843817391	10.7123375513	8.2795852092
57	H	3.0163817391	9.8552375513	8.2524852092
58	C	1.8924817391	9.6218375513	6.6320852092
59	H	2.3701817391	10.3354375513	6.1606852092
60	H	0.9402817391	9.7258375513	6.4242852092
61	C	2.3616817391	8.2636375513	6.0387852092
62	C	2.1152817391	8.1310375513	4.6716852092
63	H	1.7542817391	8.8601375513	4.2005852092
64	C	2.3827817391	6.9718375513	3.9950852092
65	H	2.1922817391	6.9082375513	3.0768852092
66	C	2.9318817391	5.8989375513	4.6613852092
67	H	3.1127817391	5.0969375513	4.2055852092
68	C	3.2123817391	6.0158375513	6.0044852092

69	H	3.5996817391	5.2865375513	6.4546852092
70	C	2.9404817391	7.1803375513	6.7165852092
71	C	3.2066817391	7.2166375513	8.1830852092
72	C	1.9975817391	7.4708375513	9.0160852092
73	C	1.4940817391	6.4817375513	9.8499852092
74	H	1.9364817391	5.6549375513	9.9084852092
75	C	0.3550817391	6.6960375513	10.5936852092
76	H	0.0210817391	6.0163375513	11.1503852092
77	C	-0.2904182609	7.9032375513	10.5202852092
78	H	-1.0690182609	8.0503375513	11.0253852092
79	C	0.2003817391	8.8983375513	9.7087852092
80	H	-0.2406182609	9.7272375513	9.6786852092
81	C	1.3436817391	8.7003375513	8.9280852092
82	C	5.5590817391	4.3183375513	8.8591852092
83	H	5.9340817391	3.4449375513	8.6208852092
84	H	4.6019817391	4.3020375513	8.6478852092
85	C	5.7259817391	4.5354375513	10.2683852092
86	H	5.2481817391	3.8218375513	10.7397852092
87	H	6.6780817391	4.4314375513	10.4761852092
88	C	5.2566817391	5.8936375513	10.8616852092
89	C	5.5030817391	6.0262375513	12.2287852092
90	H	5.8640817391	5.2971375513	12.6998852092
91	C	5.2355817391	7.1854375513	12.9053852092
92	H	5.4260817391	7.2490375513	13.8235852092
93	C	4.6865817391	8.2583375513	12.2390852092
94	H	4.5056817391	9.0603375513	12.6947852092
95	C	4.4059817391	8.1414375513	10.8959852092
96	H	4.0186817391	8.8707375513	10.4457852092
97	C	4.6779817391	6.9769375513	10.1838852092
98	C	4.4116817391	6.9406375513	8.7173852092
99	C	5.6207817391	6.6864375513	7.8843852092
100	C	6.1243817391	7.6755375513	7.0504852092
101	H	5.6818817391	8.5023375513	6.9919852092
102	C	7.2632817391	7.4612375513	6.3067852092
103	H	7.5972817391	8.1409375513	5.7500852092
104	C	7.9088817391	6.2540375513	6.3801852092
105	H	8.6873817391	6.1069375513	5.8750852092
106	C	7.4179817391	5.2589375513	7.1916852092
107	H	7.8590817391	4.4300375513	7.2217852092
108	C	6.2747817391	5.4569375513	7.9723852092
